# Reciprocal crosstalk between jasmonate and salicylate defence-signalling pathways modulates plant volatile emission and herbivore host-selection behaviour

**DOI:** 10.1093/jxb/eru181

**Published:** 2014-04-23

**Authors:** Jianing Wei, Joop J. A. van Loon, Rieta Gols, Tila R. Menzel, Na Li, Le Kang, Marcel Dicke

**Affiliations:** ^1^Laboratory of Entomology, Wageningen University, P.O. Box 8031, 6700 EH, Wageningen, The Netherlands; ^2^State Key Laboratory of Integrated Management of Pest Insects and Rodents, Institute of Zoology, Chinese Academy of Sciences, Beijing 100080, P. R. China

**Keywords:** Dose effect, herbivore behaviour, induced plant defence, phytohormonal signalling, spider mite, temporal effect.

## Abstract

The jasmonic acid (JA) and salicylic acid (SA) signalling pathways, which mediate induced plant defence responses, can express negative crosstalk. Limited knowledge is available on the effects of this crosstalk on host-plant selection behaviour of herbivores. We report on temporal and dosage effects of such crosstalk on host preference and oviposition-site selection behaviour of the herbivorous spider mite *Tetranychus urticae* towards Lima bean (*Phaseolus lunatus*) plants, including underlying mechanisms. Behavioural observations reveal a dynamic temporal response of mites to single or combined applications of JA and SA to the plant, including attraction and repellence, and an antagonistic interaction between SA- and JA-mediated plant responses. Dose-response experiments show that concentrations of 0.001mM and higher of one phytohormone can neutralize the repellent effect of a 1mM application of the other phytohormone on herbivore behaviour. Moreover, antagonism between the two signal-transduction pathways affects phytohormone-induced volatile emission. Our multidisciplinary study reveals the dynamic plant phenotype that is modulated by subtle changes in relative phytohormonal titres and consequences for the dynamic host-plant selection by an herbivore. The longer-term effects on plant–herbivore interactions deserve further investigation.

## Summary

Low-dose crosstalk between jasmonic acid and salicylic acid signalling pathways results in a dynamic plant phenotype in terms of plant volatile emission and behavioural responses of an herbivorous spider mite.

## Introduction

Plants respond to herbivory with phenotypic changes such as the production of digestibility reducers or the biosynthesis of complex blends of volatiles (Kessler and Baldwin, 2002; Heil, 2008; Dicke *et al.*, 2009). These plant responses are regulated by signalling pathways such as the octadecanoid, the shikimic acid, and the ethylene signal-transduction pathways (Dicke *et al.*, 2009; Pieterse *et al.*, 2009; Dicke and Baldwin, 2010; Atkinson and Urwin, 2012; Pieterse *et al.*, 2012; Thaler *et al.*, 2012). Crosstalk between signal-transduction pathways allows for a complex signalling network that mediates the fine-tuning of plant defences (Kessler and Baldwin, 2002; Dicke *et al.*, 2009; Pieterse *et al.*, 2009; Pieterse *et al.*, 2012; Thaler *et al.*, 2012). Plant hormones are major components of those pathways and regulate differential defence responses to specific types of attackers (Pieterse *et al.*, 2009; Leon-Reyes *et al.*, 2010; Thaler *et al.*, 2012). Generally, the phytohormones jasmonic acid (JA) and ethylene (ET) are responsible for elicitation of defences against herbivores and necrotrophic pathogens (Kessler and Baldwin, 2002; Schmelz *et al.*, 2003; von Dahl and Baldwin, 2007; Pieterse *et al.*, 2009; Wei *et al.*, 2011; Thaler *et al.*, 2012), whereas salicylic acid (SA) is predominantly involved in defence against phloem-sap-sucking insects and biotrophic pathogens (Spoel *et al.*, 2007; Pieterse *et al.*, 2009; Zhang *et al.*, 2009; Thaler *et al.*, 2010; Thaler *et al.*, 2012). Moreover, it is becoming clear that many attackers induce more than one phytohormonal pathway (e.g. De Vos *et al.*, 2005).

The SA- and JA-pathways can exhibit negative crosstalk that has been elucidated especially at the transcriptional level. Reported effects of SA on JA-dependent signalling are considerable (Spoel *et al.*, 2007; Koornneef *et al.*, 2008; Pieterse *et al.*, 2009; Thaler *et al.*, 2012). For instance, silverleaf whiteflies use SA–JA crosstalk to activate the SA pathway and consequently suppress JA-mediated defence, which accelerates their development (Zarate *et al.* 2007). In contrast, interference with SA-dependent signalling by JA was less pronounced (Kunkel and Brooks, 2002; Beckers and Spoel, 2006; Mur *et al.*, 2006). The consequences of SA–JA crosstalk for plant–herbivore interactions have been mostly investigated in terms of their effects on induced resistance (reviewed by Thaler *et al.*, 2012). However, the phytohormones JA and SA are also known to regulate the production of plant volatiles (Dicke *et al.*, 1999; Ozawa *et al.*, 2000; Lou *et al.*, 2005). Herbivory-induced plant volatiles (HIPVs) play vital roles in enabling herbivores and their natural enemies to locate their food from a distance (Dicke *et al.*, 1990; Turlings *et al.*, 1995; Bruce *et al.*, 2005; Wei *et al.*, 2007; Dicke and Baldwin, 2010; Bruce and Pickett, 2011). Although a few studies have explored such negative SA–JA crosstalk in plant–herbivore–natural enemy interactions (Zhang *et al.*, 2009; Thaler *et al.*, 2010), to date it is largely unknown how SA–JA negative crosstalk affects host-plant selection behaviour of herbivores. Moreover, the extent to which this SA–JA crosstalk is reciprocal and how this affects herbivore host-plant selection has received limited attention (Dicke *et al.*, 2009; Thaler *et al.*, 2012).

HIPVs are important mediators of plant–herbivore interactions (Dicke and Baldwin, 2010), such as the well-studied interaction between Lima bean plants (*Phaseolus lunatus* L.) and spider mites (*Tetranychus urticae* Koch). For instance, *T. urticae* avoided the odours of conspecific-infested bean plants compared with uninfested control plants (Dicke, 1986; Harrison and Karban, 1986). Olfactometer experiments with cucumber plants showed that *T. urticae* preferred the odours of plants infested with conspecifics (*T. urticae*), but strongly avoided plants infested by thrips (*Frankliniella occidentalis*) (Pallini *et al.*, 1997). These differential behavioural responses might be caused by differences in duration of infestation until the behavioural tests (temporal effects), or differences in herbivore species or density (dose effects), or plant species. Because plant responses to herbivory include JA- and SA-mediated responses and these phytohormones crosstalk (Thaler *et al.*, 2012), we here address the effects of these phytohormones, singly or combined, on plant-mediated host selection by the spider mite *T. urticae* as affected by different doses and temporal patterns.

We explore the reciprocal interactions between JA- and SA-signal-transduction pathways in Lima bean plants for their effects on feeding-site selection by the spider mite *T. urticae* as well as the consequences for spider-mite oviposition site selection. We show that reciprocally antagonistic crosstalk between the JA- and SA-signalling pathways modulates spider-mite preference and we connect this to volatile biosynthesis of Lima bean plants and the transcription of a few selected genes. Our data provide strong evidence that reciprocal antagonism between SA- and JA-signalling pathways affects plant volatile emission and herbivore host-selection behaviour.

## Materials and methods

### Rearing plants and mites

Lima bean plants, *Phaseolus lunatus* L. cv. Sieva, were grown in a greenhouse compartment at 25±5 °C, 50–70% R.H., and a photoperiod of 16L:8D. Lima bean plants were used in experiments when their two primary leaves had fully expanded, i.e. 10–15 d after sowing. The two-spotted spider mite, *Tetranychus urticae* Koch (Acari: Tetranychidae), was reared on Lima bean plants in a greenhouse compartment under the same conditions as those for plant growth. Adult female *T. urticae*, which had hatched from the same cohort of eggs 10 d before and were observed to lay eggs (Zhang *et al.*, 2009), were used in all experiments.

### Behavioural bioassay

To investigate whether spider-mite behaviour was affected by the odours from different sources, two-choice experiments were carried out. To this end, a trapezoid-shaped bridge (length long side: 3cm, pillar: 1cm, width: 0.5cm, thickness: 1mm) was positioned such that it connected two Lima bean leaf sections, lying on a wet cotton-wool disk in an open Petri dish (see Supplementary Fig. S1 available at *JXB* online). A spider mite was individually placed at the middle of the bridge and allowed to walk to either side, where it had to make a choice by mounting either leaf disk (2cm in diameter) cut from leaves from an intact Lima bean plant just before the experiment. Once the mite entered one of the leaf sections, its choice was recorded as “first choice”. The observation lasted maximally 15min for each female mite. The positions of the two odour sources (leaf disks) were alternated among replicates. On every experimental day, 20 individual mites were observed for each odour source combination (see section on JA and SA treatment below). After observation of the first choice, these set-ups were kept in a growth chamber at 22±2 °C, 50–60% R.H. and a photoperiod of 16L:8D. After 24h post inoculation (hpi), the position of mites and number of eggs deposited on each leaf disk were recorded. In total, each test was replicated on three different days and 60 mites were used for each odour-source combination.

### JA and SA treatment

Plants were treated with a JA or SA solution. In all cases 1.25ml was sprayed per leaf. (i) Time course of the effect of JA or SA on mite choice behaviour. Solutions of 1mM JA ((±)-jasmonic acid, Sigma-Aldrich, purity > 97%, dissolved in tap water by vigorously shaking) or SA (salicylic acid, Sigma-Aldrich, purity > 98%, diluted in 1ml ethanol (99%), then further diluted in tap water) were sprayed on the Lima bean leaves, and incubated for 24, 48, 72, 96, and 120h before behavioural experiments. At the same time, on control plants, tap water or a 1% ethanol solution in tap water, were sprayed on the Lima bean plants and incubated for the same time as the respective treatments. (ii) Time course of the effect of combined JA and SA applications on mite choice behaviour. A solution of 1mM JA was sprayed on the Lima bean plants and allowed to dry for 30min, followed by spraying of a 1mM SA solution and then the plants were incubated for 24, 48, and 72h before being used in the experiments. On control plants, tap water and a solution of 1% ethanol in tap water were sprayed and these plants were incubated for the same time as those of the combined JA / SA treatment. (iii) Reciprocal antagonism between SA and JA signalling pathways in Lima bean. To assess the effect of SA on plants treated with 1mM JA, solutions of 1mM JA were sprayed on the Lima bean leaves and these leaves were subsequently sprayed with a solution of 0, 0.0001, 0.001, 0.01, 0.1, or 1mM SA and all treated plants were incubated for 24h before experiments. Tap water and solutions of 0.0001%, 0.001% 0.01%, 0.1%, or 1% ethanol in tap water were sprayed on plants that served as controls and the plants were then incubated for 24h. For the experiments on the effect of JA application on plants treated with SA, plants were sprayed with a solution of 1mM SA or 1% ethanol (control). All treated plants were incubated for 24h. After that, solutions of 0, 0.0001, 0.001, 0.01, 0.1, or 1mM JA were sprayed on SA-treated plants; water was sprayed on the ethanol-treated control plants. All treated plants were incubated for another 24h before being used in the experiments. The difference in timing to study the effect of SA on JA-induction versus the effect of JA or SA induction were instigated by the results of the first experiment and will be explained in the Results section. (iv) Persistence of the SA-mediated antagonism to the repellent effect of 1mM JA treatment. Solutions of 1mM SA or 0.001mM SA were sprayed on Lima bean leaves and the treated plants were incubated for 24h, 48h, or 72h. Control plants were sprayed with solutions of 0.001% or 1% ethanol and were incubated for the same time period. Then the SA-treated plants were sprayed with 1mM JA and the control plants with tap water and all plants were incubated for another 24h before being used in the experiments. As negative control, plants were sprayed with solutions of 0.001% or 1% ethanol and they were incubated for 24h. Half of these plants were sprayed with 1mM JA and were incubated for another 24h. The other half of the plants were sprayed with tap water (control plants) and they were incubated for 24h before behavioural experiments.

In volatile collection experiments we investigated the two extremes of SA–JA crosstalk that were recorded in the behavioural experiments. We investigated: (i) The effect of SA on JA-induced volatile emission. A solution of 0.001mM SA or 0.001% ethanol was sprayed on leaves of Lima bean plants and these plants were incubated for 24h. Then 1.0mM JA was applied and headspace volatiles of treatment and control plants were collected 24h later. (ii) The effect of JA on SA-induced volatile production. Lima bean plants were sprayed with a solution of 1mM SA and then they were incubated for 24h. Half of these plants were sprayed with 0.001mM JA and were incubated for another 24h. Another half were sprayed with tap water and served as control plants; the plants were incubated for 24h before volatile collection.

For gene expression experiments the Lima bean plants were treated with 1mM JA for 24h, or they were treated with 0.001mM SA for 24h and were then treated with 1mM JA for another 24h before RNA extraction. Control plants relative to JA treatment were sprayed with water and control plants relative to SA treatment were treated with 0.001% ethanol for 24h plus water for another 24h before RNA extraction.

### Headspace volatile collection

Volatiles emitted by Lima bean plants were collected with a dynamic headspace collection system (Loivamaki *et al.*, 2008). For details see the online Supplementary data (available at *JXB* online).

### Chemical analysis of headspace volatiles

Headspace samples were analysed as described previously (Zhang *et al.*, 2009). For details see the online Supplementary data.

### Quantitative real-time PCR

Total RNA extraction and purification were done as described in the handbook of RNeasy Plant Mini Kit (Qiagen Group, Valencia, CA, USA). cDNA synthesis was performed as reported previously (Zheng *et al.*, 2007). To quantify *lipoxygenase* (*LOX*), *P. lunatus Ocimene Synthase* (*PlOS*), and *pathogenesis-related protein 2* (*PR-2* (β-1,3-glucanase)) transcript levels, real-time quantitative RT-PCR was performed in a Rotor-Gene 6000 machine (Corbett Research) with a 72-well rotor (Zhang *et al.*, 2009). For details see the online Supplementary data.

### Statistical analysis

A χ^2^ test was used to analyse mite preference behaviour. A paired two-tailed *t*-test was employed to assess the significance of differences in average number of eggs deposited on treatment and control leaf disks at each time point. The gene expression levels relative to *PlACT1* (fold changes) were log-transformed and statistically analysed by ANOVA following Tukey’s honestly significant difference (HSD) test. Principal component analysis was used to reveal which volatile compounds are important for the separation of volatile blends emitted by plants treated with JA alone or a combination of JA and SA. Similarly, we analysed the volatiles emitted by plants treated with SA alone and those treated with SA followed by JA. We used the PLS-DA (Projection to Latent Structures Discriminant Analysis) extension of the SIMCA P+ 12.0 software program, (Umetrics AB, Umeå, Sweden). For more details see the online Supplementary data.

## Results

### Time course in the effect of phytohormone treatments on mite choice behaviour

**Fig. 1.**
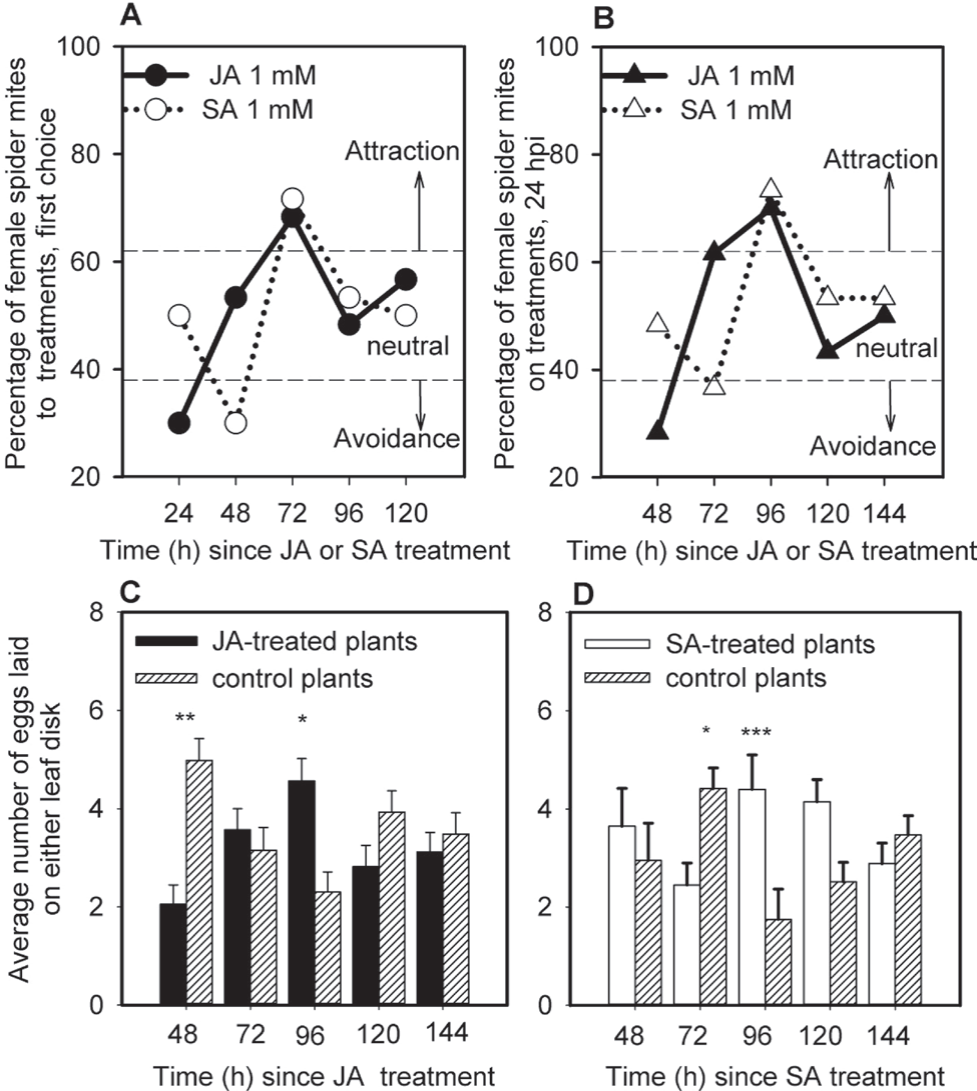
Time course of the effect of JA- or SA-treatment on female mite choice behaviour and oviposition on leaf disks cut from Lima bean plants just before the experiment. (A) Percentage of mites choosing leaf disks treated with 1mM JA or 1mM SA as first choice within 15min. (B) Distribution of mites over JA- or SA-treated leaf disks 24h post-inoculation (hpi) in the arena. (C) Number of eggs laid by female spider mites on JA-treated Lima bean disk versus that on control leaf disk (mean±SE) during 24h. (D) Number of eggs laid by mites on SA-treated Lima bean disks versus that on control leaf disk (mean±SE) during 24h. These experiments were repeated three times at three different days, each with 20 mites. Thus, in total 60 mites were individually studied for each treatment and time point (*n*=60; data of the individual replicates are provided in Table S1). In A and B data points located in areas above or below dashed lines indicated by arrows designated ‘attraction’ or ‘avoidance’ indicate a choice distribution significantly different from a 50:50 distribution (α=0.05, χ^2^ test); (see Supplementary Table S1 for details of the statistical analyses). In C and D, data within a time point are analysed with a paired two-tailed *t*-test, * *P*<0.05, ** *P*<0.01, *** *P*<0.001 (see Supplementary Table S2 for details of the statistical analyses). Before parametric analysis, oviposition data were log (x+1) transformed to correct for heterogeneity of variances.

To investigate whether the preference of spider mites depends on the time elapsed since application of JA or SA to Lima bean leaves, the initial selection behaviour of spider mites, i.e. their choice before contacting the leaf disks, was observed in two-choice experiments. When offered a choice between leaf disks taken from plants treated with 1.0mM JA and control (treated with water) plants, the mites’ choice changed from initial avoidance at 24h post application (hpa) to attraction 72 hpa to no effect at 96 and 120 hpa (Fig. 1A). Similar temporal dynamics were observed for the effects of SA treatment; however, avoidance was observed only at 48 hpa. The distribution of mites over treatment and control at 24h post inoculation (hpi) was similar to the first choice of the mites for both phytohormones (Fig. 1B). The number of eggs deposited on disks from phytohormone-treated and control plants correlated with the distribution of mites over the two disks observed at every time point (Fig. 1C and 1D). The application of 1mM JA or SA did not result in a differential choice of female spider mites for phytohormone-treated disks over control leaf disks within 1 hpa (see Supplementary Fig. S2). Therefore, the effects of JA and SA treatment on the behaviour of the spider mites are not due to the presence of JA or SA itself. These results were used as reference for investigating possible crosstalk between the JA- and SA-signal-transduction pathways and its effect on spider-mite host-selection behaviour.

### Effects of combined JA and SA application on mite choice behaviour

**Fig. 2.**
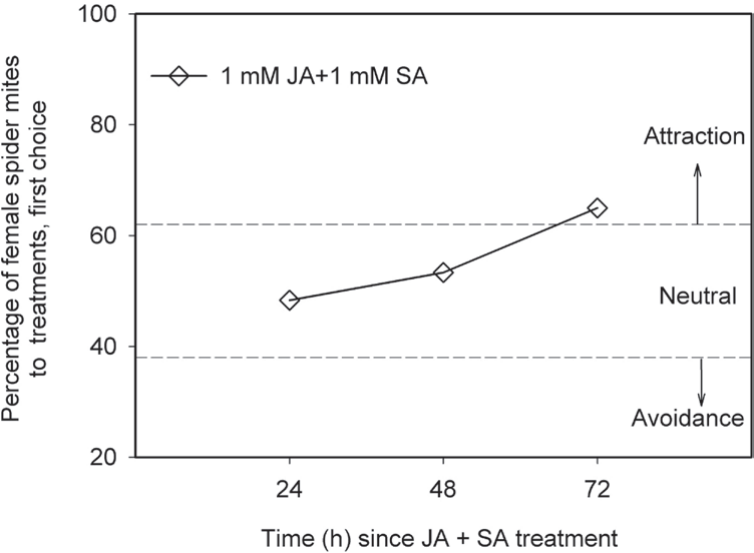
Time course of effects of combined 1mM JA + 1mM SA application versus control on the preference of female spider mites for leaf disks cut from Lima bean plants just before the experiment. These experiments were repeated three times at three different days, each with 20 mites; thus, a total of 60 mites were individually used in each treatment (*n*=60; data of the individual replicates are provided in Table S1). Data points located in areas above or below dashed lines indicated by arrows designated ‘attraction’ or ‘avoidance’ indicate a choice distribution significantly different from 50:50 (α=0.05, χ^2^ test) (see Supplementary Table S1 for details of the statistical analyses).

Combined JA+SA application (both at 1mM) did not result in a preference for treatment or control plants at 24 hpa or 48 hpa, but leaf disks from JA+SA-treated plants were preferred over control plants at 72 hpa (Fig. 2). In addition, the distributions of mites and their eggs on leaf disks of JA+SA treatment versus control were consistent with initial preference of the mites (see Supplementary Fig. S3 available at *JXB* online). The combined JA+SA application did not yield an avoidance response and the response was different from the response to single phytohormone application at 24 and 48 hpa (Fig. 1A and 2), suggesting that there were antagonistic effects of SA on JA and/or vice versa. The attraction at 72 hpa is similar to the effect of single phytohormone treatments, suggesting that there is no negative crosstalk but also no additive positive effect of an interaction between effects caused by SA and JA at this time point.

### Reciprocal antagonism between SA- and JA signalling pathways in Lima bean modulates mite choice behaviour

**Fig. 3.**
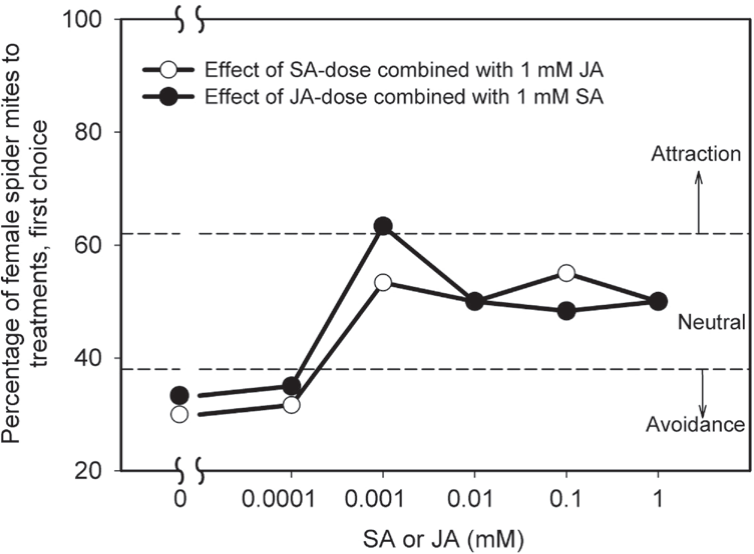
First choice of female spider mites in 15 min-two-choice behavioural assays offering leaf disks cut from Lima bean plants just before the experiment, as affected by combined application of JA (1mM) and SA at five different concentrations (open symbols) and SA (1mM) and JA at 5 different concentrations (filled symbols) versus control disks. These experiments were repeated three times at three different days, each with 20 mites. Thus, in total 60 mites were individually studied for each treatment and time point (*n*=60; data of the individual replicates are provided in Table S1). Data points located in areas above or below stippled lines indicated by arrows designated ‘attraction’ or ‘avoidance’ indicate a choice distribution significantly different from 50:50 (α=0.05, χ^2^ test) (see Supplementary Table S1 for details of the statistical analyses).

To investigate the effects of SA-dose on JA–SA crosstalk, SA was applied to Lima bean leaves in 5 concentrations in combination with a 1.0mM JA treatment. After 24h, leaf disks from plants that had been exposed to these combined treatments as well as the corresponding disks from control plants were used in choice experiments to assess whether spider mite avoidance of 1mM JA-induced leaf disks at 24 hpa was antagonized by different dosages of SA. Treating plants with 0.0001mM SA had no antagonistic effect on the repellence induced by 1mM JA, but 0.001mM and higher concentrations neutralized JA-induced repellence (Fig. 3). Distribution of mites and their eggs at 24 hpi were consistent with initial mite preference (see Supplementary Fig. S4).

The effect of 1.0mM SA is that the spider mites are repelled after 48h of incubation. To investigate the dose effect of JA on this SA-mediated effect, 1.0mM SA was applied to Lima bean leaves and incubated for 24h. Then, JA was applied to these SA-treated Lima bean plants in one of five different concentrations and incubated for another 24h. Spider mites were subsequently offered a choice between leaf disks taken from treated and control plants to assess whether SA-induced repellence to spider mites at 48h after 1.0mM SA application was antagonized by different doses of JA. Concentrations of JA below 0.001mM had no effect on the repellence induced by 1mM SA, whereas all higher doses resulted in either attraction or no preference (Fig. 3). Distribution of mites and eggs at 24 hpi was consistent with initial preference (see Supplementary Fig. S4 available at *JXB* online). In conclusion, very low doses of SA or JA can antagonize the repellent effects on spider mites that are induced by treatment with the other phytohormone.

### Duration of antagonistic effect of SA on JA-induced avoidance by mites

**Fig. 4.**
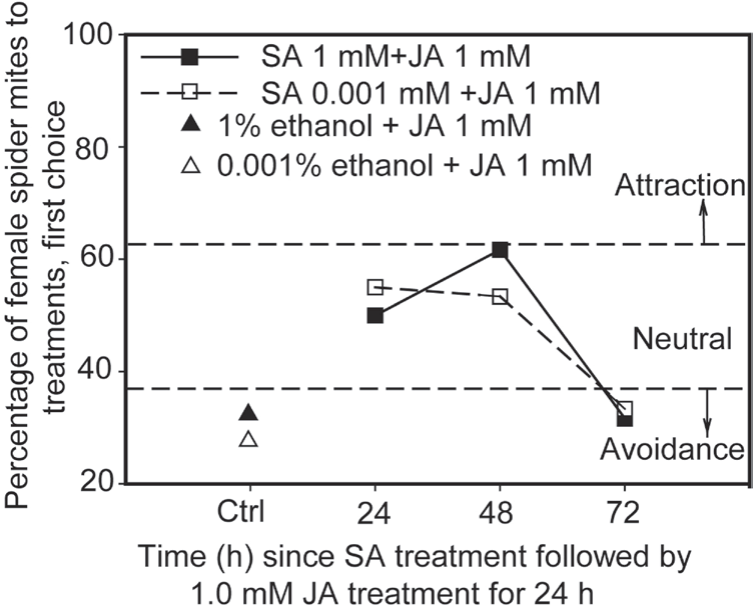
Persistence of the SA-mediated antagonistic effect on JA-induced repellency to spider mites: first choice of female spider mites in 15 min-two-choice behavioural assays offering leaf disks cut from Lima bean plants just before the experiment. These experiments were repeated three times at three different days, each with 20 mites; thus, a total of 60 mites were individually used in each treatment (*n*=60; data of the individual replicates are provided in Table S1). Ctrl: the plants were sprayed with the solutions of either 0.001% or 1% ethanol as negative controls for 0.001mM SA- and 1mM SA-treatments respectively. Data points located in areas above or below dashed lines indicated by arrows designated ‘attraction’ or ‘avoidance’ indicate a choice distribution significantly different from 50:50 (α=0.05, χ^2^ test) (see Supplementary Table S1 for details of the statistical analyses).

To investigate the persistence of the SA-mediated antagonism to the repellent effect resulting from 1mM JA treatment, we applied the lowest effective dose of SA (0.001mM) or 1mM SA to Lima bean leaves at several time points preceding 1.0mM JA application. The negative control experiments were done by applying 0.001% ethanol (see methods section) or 1% ethanol on Lima bean leaves 24h before 1.0mM JA application. Antagonism by SA on JA-induced repellency was observed when either SA-dose was applied up to 48h before JA treatment (Fig. 4). When the time interval between the SA and JA application exceeded 48h, no antagonism by SA was observed (Fig. 4). The observed persistence of the effect of SA on JA-treatment was similar for the 0.001mM and 1mM SA doses (Fig. 4). The distribution of mites and eggs at 24 hpi was consistent with initial preference of the mites (see Supplementary Fig. S5).

### Reciprocal antagonism between SA and JA signalling pathways in Lima bean affects phytohormone-induced volatile production

**Fig. 5.**
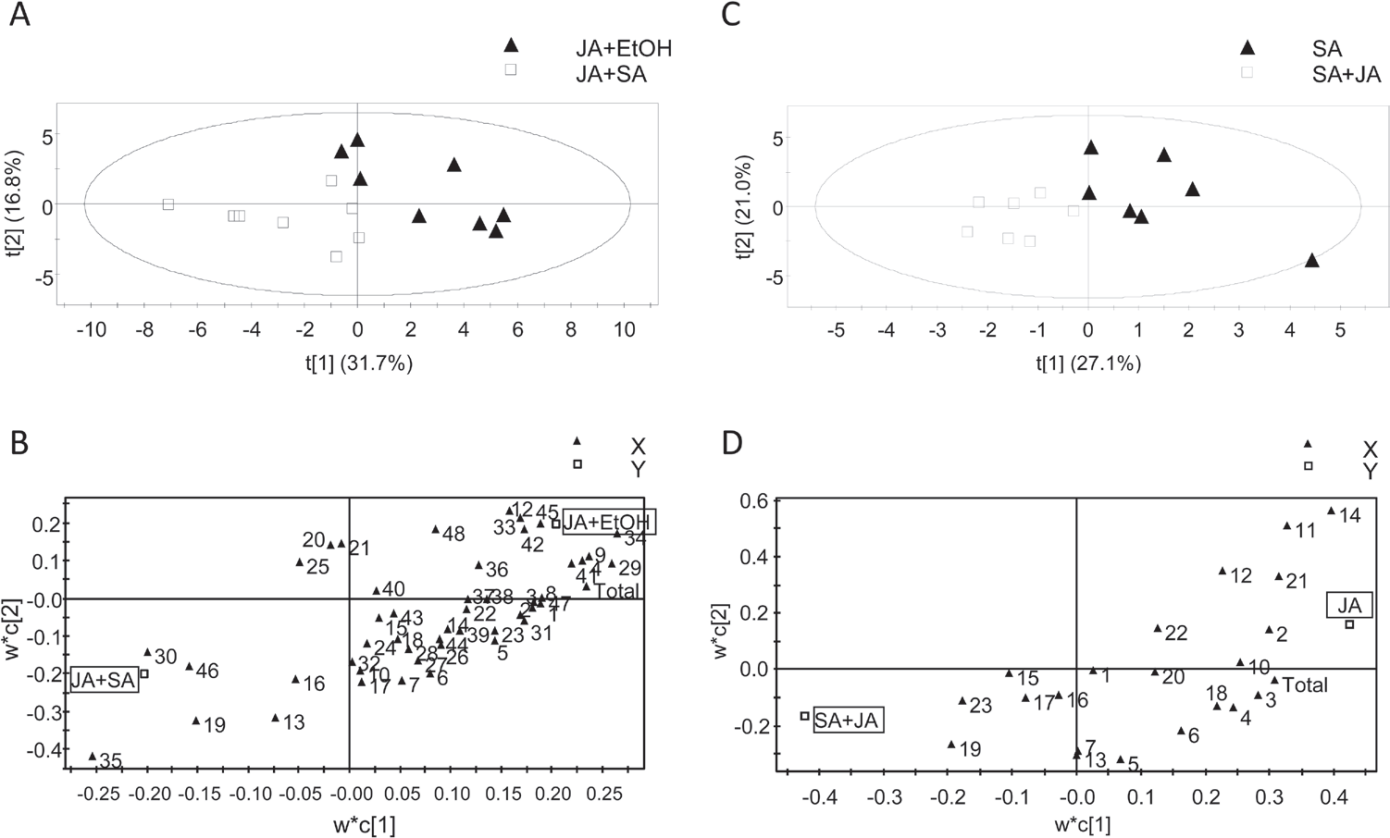
Reciprocal antagonism between SA and JA signalling pathways in Lima bean affects induced volatile emission. Projection to latent structures-discriminant analysis (PLS-DA) of volatile emissions. Score plot (A and C) and loading plot (B and D) of the first two principal components with the explained variance in brackets. The ellipse defines the Hotelling’s T2 confidence region (95%). The triangles in the loading plots represent volatile emission of individual compounds where numbers refer to compound numbers as indicated in Supplementary Table S3. (A, B) Comparison of volatile emission by Lima bean plants treated with 0.001mM SA plus 1.0mM JA and from plants treated with EtOH (0.001% ethanol) plus 1.0mM JA (*n=*8). (C, D) Comparison volatile emission by plants treated with 1mM SA plus 0.001mM JA and from plants treated with 1mM SA plus water (*n=*7). For details on volatile profiles, see Supplementary Tables S3 and S4 (available at *JXB* online). ‘Total’ is total emission of all compounds together.

Based on the observed shifts in behavioural preferences, we hypothesized that crosstalk between the JA and SA signalling pathways affects the amount and/or composition of volatile emissions in Lima bean plants. To investigate the effect of SA on JA-induced volatile production, we addressed the effect of the lowest effective SA dose. We applied 0.001mM SA or 0.001% ethanol (control) to Lima bean plants and incubated them for 24h. Subsequently, 1.0mM JA was applied and the headspace volatiles of treatment and control plants were collected 24h later. The headspace analyses show a significant difference in the total amount of volatiles released between treatment and control plants (two-tailed paired *t*-test: *t*=3.021, *df*=14, *P*=0.0092; see Supplementary Table S3 at *JXB* online for details on headspace composition). PLS-DA resulted in a model with 5 significant principal components (PCs; model statistics: *R* ^2^X=0.77, *R* ^2^Y=0.99 and *Q* ^2^=0.88) of which the first two explained 48.5% of the variance and clearly separated the data points for the two treatments into two groups according to treatment (Fig 5A). SA treatment antagonized the emission of volatiles by JA-treated plants (see Supplementary Table S1) and most of the compounds in the loading plot are located on the right side of the figure (Fig. 5B). Also the total emission was included in the analysis and this variable also strongly contributes to the separation of the samples according to plant treatment (being in the top right corner of the loading plot). These data show that a very low dose of SA is able to down-regulate most of the JA-induced volatile emission by Lima bean plants.

Conversely, to investigate the effect of JA on SA-induced volatile production, we also addressed the lowest effective JA dose. We collected the headspace volatiles from plants that had been treated with 1mM SA for 24h plus a subsequent exposure to 0.001mM JA for another 24h; as controls we collected the headspace from plants that had been treated with 1mM SA for 24h, plus a subsequent exposure to water for another 24h. The SA-induced volatile blend is characterized by a significantly lower number of compounds than the JA-induced volatile blend and the compounds common to both blends occurred in significantly lower amounts in the former blend (see Supplementary Tables S4 and S5). PLS-DA resulted in a model with two significant PCs (model statistics: *R* ^2^X=0.48, *R* ^2^Y=0.76, and *Q* ^2^=0.23), which explained 48% of the variance (Fig. 5C). Also here, the majority of the compounds, as well as the total emission, contribute to the separation of the samples according to treatment. Thus, a low dose of JA down-regulated the SA-induced volatile emission by Lima bean plants. Control untreated plants emitted minor amounts of volatiles (see Supplementary Table S6).

### Effect of SA on JA-induced gene expression

By using quantitative RT-PCR, we quantified the transcript levels of three genes involved in plant defence, *Lipoxygenase* (*LOX*), *P. lunatus Ocimene Synthase* (*PlOS*), and *Pathogenesis-Related protein 2* (*PR-2* (β-1,3-glucanase)), in plants treated with 1mM JA or 1mM JA + 0.001mM SA, compared with control plants. *LOX* is a major JA-inducible gene in the octadecanoid signalling pathway. The JA-induced *LOX*-transcript levels were similar for both plant groups (two-tailed sample *t*-test: *t=*0.789, *df=*10, *P=*0.449; Supplementary Fig. S8A available at *JXB* online), whereas the *PlOS-*transcript level gene was significantly down-regulated by SA treatment (*t=*2.232, *df=*10, *P=*0.049; Supplementary Fig. S8B). This correlates well with the observed effects of SA on the emission of (*E*)-β-ocimene (see Supplementary Table S1), whose biosynthesis is regulated by *PlOS.* Moreover the *PR-2* transcript level was up-regulated by JA treatment and marginally significantly even further by additional SA treatment (*t=*1.949, *df=*10, *P=*0.079; Supplementary Fig. S8C).

## Discussion

JA and SA are two of the most important phytohormones involved in the induction of plant defence against herbivores and pathogens (Heil and Walters, 2009; Pieterse *et al.*, 2009; Thaler *et al.*, 2012). The temporal dynamics and level of induction of JA and SA vary with the attacker that damages the plant (De Vos *et al.*, 2005). Individual attackers induce different combinations of phytohormones with different temporal and dosage patterns (Ozawa *et al.*, 2000; Engelberth *et al.*, 2001; Kessler and Baldwin, 2002; Pieterse *et al.*, 2012). Moreover, plants are seldom infested by a single attacker and multiple attack may add to the complexity of the dynamics of phytohormonal patterns. Multiple attack and the resulting interplay between phytohormones may alter plant-mediated interactions between attackers as well as their natural enemies (Bostock, 1999; Voelckel and Baldwin, 2004; Zhang *et al.*, 2009; Rodriguez-Saona *et al.*, 2010; Erb *et al.*, 2011; Bukovinszky *et al.*, 2012; Ponzio *et al.*, 2013; Stam *et al.* 2014).

The effects of crosstalk between JA and SA signal-transduction pathways have been extensively explored at the molecular level and in the context of plant resistance to attackers (Cui *et al.*, 2005; Beckers and Spoel, 2006; Koornneef *et al.*, 2008; Thaler *et al.*, 2010; Thaler *et al.*, 2012). However, so far investigations on the effects of multiple attackers in the context of temporal dynamics and different doses of the induced phytohormonal patterns on herbivore host-plant selection as well as the consequences for their reproduction have received virtually no attention (Thaler *et al.*, 2012). Here, we addressed the effects of JA and SA singly and in combination with different temporal patterns and doses to understand the effects of the interaction between JA and SA in Lima bean plants on host selection behaviour of the spider mite *T. urticae* on plant volatile emission. Our data show that the application of a single phytohormone has a temporally dynamic effect on host selection by *T. urticae.* More importantly, SA negatively crosstalks to JA and vice versa, and this was observed for relatively low doses of the interfering phytohormone. For the behavioural experiments with the small mites (ca 0.7mm in length) we have used an often-employed two-choice setup consisting of leaf disks that were freshly cut from the treated and control plants (Gols *et al.*, 2003; Leon-Reyes *et al.*, 2009; Zhang *et al.*, 2009). Headspace analysis of leaf disks showed that cutting of leaf disks results in a short-lasting transient emission of a few green leaf volatiles, but this treatment did not result in JA or SA-induced volatile compounds in leaf disks from control plants (see Supplementary Figs S6 and S7), supporting the conclusion that the mite choices in our studies are due to the phytohormonal treatments and not to punching the leaf disks, which was done for both treatment and control alike.

A large body of research has shown that host-plant preference by herbivores is affected either negatively or positively by exogenous application of JA or methyl jasmonate (MeJA). For instance, a 1mM JA treatment of *Brassica oleracea* plants resulted in avoidance by *Pieris rapae* and *P. brassicae* butterflies at 24 hpa (Bruinsma *et al.*, 2007), whereas JA-treated *B. oleracea* plants were preferred for oviposition over controls by the diamondback moth, *Plutella xylostella* (Lu *et al.*, 2004). The abundance of herbivores was significantly reduced by one or two early-season treatments of field-grown wild tobacco or tomato plants with MeJA or JA (Kessler and Baldwin, 2001; Thaler *et al.*, 2001), indicating that the effect of exogenous application of JA can persist for weeks. However, how a single application of different dosages of JA affects herbivore food selection, how the effect of application develops over time, and what interaction occurs with SA application had thus far not been studied. Here, we found that JA-induced host-plant selection by spider mites changes markedly over a 72 h-time period. The choice behaviour of the mites was affected by cues that they perceived before making contact with the leaf disks. Because JA application results in the emission of HIPVs that function as attractant or repellent to adult herbivores (Dicke *et al.*, 1999; Ozawa *et al.*, 2000; van Dam *et al.*, 2000; Gols *et al.*, 2003; Halitschke *et al.*, 2008), the temporal dynamics of HIPV-emission probably caused the shifts in female spider-mite host-plant preference.

SA application to plants is known to elicit direct-defence responses (Ozawa *et al.*, 2000; Thaler *et al.*, 2002). We previously showed that spider mites prefer to feed on and have higher oviposition rates on 1mM SA-treated Lima bean plants as compared with uninfested plants at 6 d after SA-application (144 hpa), suggesting that SA application did not interfere with foraging behaviour and reproduction (Zhang *et al.*, 2009). In the present study, application of 1mM SA on Lima bean leaves did not result in preference at earlier time points, i.e. 96 and 120 hpa. Moreover, we found that the numbers of eggs deposited on treated and control leaf disks are consistent with the preference observed after 15min showing that preference is linked to reproductive output. The changes in the preference of mites over the 5-day period following SA-treatment must therefore be sought in the temporal dynamics of HIPV-emissions.

Abundant molecular evidence shows that the JA- and SA-signalling pathways exhibit negative crosstalk (Pieterse *et al.*, 2009; Thaler *et al.*, 2012). For instance, SA-mediated suppression of JA-responsive gene expression has been shown in *Arabidopsis thaliana*, Lima bean, tomato, and tobacco plants (Thaler *et al.*, 2002; Koornneef *et al.*, 2008; Pieterse *et al.*, 2009; Leon-Reyes *et al.*, 2010). The transcription factor WRKY70 and the defence-regulating protein NPR1 were shown to play dual roles in regulating SA-mediated activation of SA-dependent defences as well as SA-mediated suppression of JA-dependent defences (Pieterse *et al.*, 2009). Moreover, SA and MeJA treatments applied at different concentrations and time intervals and using SA-inducible *PR-1* and MeJA-inducible *PDF1.2* and *VSP2* as marker genes, revealed the molecular kinetics of SA-JA negative crosstalk in *Arabidopsis* (Koornneef *et al.*, 2008). A concentration as low as 0.0001mM SA suppressed MeJA-induced *PDF1.2* transcription in *Arabidopsis* plants and this suppression was lost between 30 and 48 hpa (Koornneef *et al.*, 2008). However, no suppressive effect of JA on transcription of the SA-responsive gene *PR-1* was found in *A. thaliana*. Our study is the first to take the temporal dynamics of SA–JA interaction to the level of volatile biosynthesis and its consequences for herbivore host-selection behaviour. We showed that the application of both a high (1mM) and a low (0.001mM) dose of SA suppressed JA-induced repellence to spider mites and JA-inducible *PlOS* gene transcription in Lima bean, which corresponds to the recorded SA-mediated reduction in JA-induced (*E*)-*β*-ocimene emission. These data further confirm the SA-mediated suppression of JA signalling.

A few studies have documented the effect of negative SA–JA crosstalk on herbivore-induced plant volatile (HIPV) emission. Simultaneous infestation of cotton plants by JA-pathway-inducing beet armyworm caterpillars (*Spodoptera exigua*) and SA-pathway-inducing whiteflies (*B. tabaci*) resulted in lower emission rates of HIPVs than from plants infested by beet armyworm caterpillars only (Rodriguez-Saona *et al.*, 2003). We previously showed that the application of 1mM SA to Lima bean can significantly suppress the emission of major spider mite-induced volatiles, such as (*E*)-β-ocimene and (*Z*)-β-ocimene, compared with plants infested with *T. urticae* only (Zhang *et al.*, 2009). In the present study, we further demonstrated that a 1000-times lower dose of SA is sufficient to suppress the emission of many JA-induced volatile compounds, suggesting that this down-regulation is highly sensitive to SA. Moreover, a low dose of JA (0.001mM) significantly suppressed the emission of SA-induced volatiles.

Although a number of studies showed that plant-mediated effects of pathogen infection on herbivorous insects affect the host-selection behaviour of herbivores (Stout *et al.*, 2006), up to date it is largely unknown whether these infestations interfere with herbivore choice behaviour through signalling crosstalk. In the present study, we mimicked the multiple interactions between herbivore and pathogen species with exogenous applications of JA and SA in different temporal patterns and doses. Our behavioural results not only demonstrate that there are antagonistic effects of SA-mediated responses on JA-mediated responses and vice versa, but also that dose and timing of combinations of phytohormone treatments affect the behavioural responses of an herbivore. Importantly, phytohormone-mediated effects of negative crosstalk on preference of herbivore result in significant consequences for spider-mite oviposition choices in all cases suggesting that female spider mites have developed behavioural strategies to use information of the plant defensive signalling network for their own benefit. Therefore, the biological and evolutionary significance of crosstalk between SA- and JA-dependent defence responses deserves further elucidation in response to actual feeding or infestation by multiple herbivores and/or pathogens.

Understanding the ecological effects of phytohormonal dynamics in response to multiple attack in terms of host-plant selection by herbivores and their natural enemies may be exploited to develop environmentally friendly ways to increase resistance of agricultural crops to combinations of pests and pathogens.

## Supplementary data

Supplementary data are available at *JXB* online.

Figure S1. Two-choice set-up to investigate odour-based attraction of female spider mites to leaf disks exposed to phytohormone treatments.

Figure S2. Short-term effect of JA or SA treatments on preference of female spider mites.

Figure S3. Time course of effects of JA + SA-treatment versus control (1% ethanol) on distribution and oviposition of female spider mites on Lima bean leaf disks.

Figure S4. Distribution and oviposition of female spider mites on Lima bean leaf disks in a two-choice situation as affected by combined application of JA (1mM) and SA at 5 different concentrations (open symbols) and SA (1mM) and JA at 5 different concentrations (filled symbols) vs. control disks.

Figure S5. Persistence of the SA-mediated antagonistic effect on JA-induced repellency to spider mites.

Figure S6. Green leaf volatiles collected from the headspace of leaf disks taken from healthy bean plants.

Figure S7. Volatile compounds released from leaf disks taken from healthy bean plant, plants treated with 1mM JA for 24h (A), and plants treated with 1mM SA for 48h (B).

Figure S8. Transcript levels of *LOX* (A), *PlOS* (B), and *PR-2* (C) relative to control (water treated) plants in Lima bean plants sprayed with 1mM JA, or 0.001mM SA plus 1mM JA.

Table S1. Statistical analyses of odour-based preferences of female spider mites to leaf disks of different treatments in all experiments.

Table S2. Statistical analyses of numbers of eggs laid by female spider mites on JA-or/ and SA-treated Lima bean disks versus that on control leaf disks.

Table S3. Absolute and relative (%) amount of volatiles released from Lima bean plants treated with 0.001mM SA for 24h and then with 1.0mM JA application for another 24h and from plants with control (0.001% ethanol treatment) and 1mM JA applications in same time interval.

Table S4. Absolute and relative (%) amount of volatiles released from Lima bean plants treated with 1mM SA for 24h and then with 0.001mM JA application for another 24h and from plants with control (tap water treatment) and 0.001mM JA applications in same time interval.

Table S5. Absolute and relative (%) amount of volatiles released from Lima bean plants treated with controls (tap water and 0.001% ethanol treatment).

Supplementary Data

## References

[CIT0001] Atkinson NJ Urwin PE 2012 The interaction of plant biotic and abiotic stresses: from genes to the field. Journal of Experimental Botany 63, 3523–3543 22467407 10.1093/jxb/ers100

[CIT0002] Beckers GJM Spoel SH 2006 Fine-tuning plant defence signalling: Salicylate versus jasmonate. Plant Biology 8, 1–10 16435264 10.1055/s-2005-872705

[CIT0003] Bostock RM 1999 Signal conflicts and synergies in induced resistance to multiple attackers. Physiological and Molecular Plant Pathology 55, 99–109

[CIT0004] Bruce TJA Pickett JA 2011 Perception of plant volatile blends by herbivorous insects—Finding the right mix. Phytochemistry 72, 1605–1611 21596403 10.1016/j.phytochem.2011.04.011

[CIT0005] Bruce TJA Wadhams LJ Woodcock CM 2005 Insect host location: a volatile situation. Trends in Plant Science 10, 269–274 15949760 10.1016/j.tplants.2005.04.003

[CIT0006] Bruinsma M Van Dam NM Van Loon JJA Dicke M 2007 Jasmonic acid-induced changes in *Brassica oleracea* affect oviposition preference of two specialist herbivores. Journal of Chemical Ecology 33, 655–668 17334923 10.1007/s10886-006-9245-2 PMC1915630

[CIT0007] Bukovinszky T Poelman EH Kamp A Hemerik L Prekatsakis G Dicke M 2012 Plants under multiple herbivory: consequences for parasitoid search behaviour and foraging efficiency. Animal Behaviour 83, 501–509

[CIT0008] Cui J Bahrami AK Pringle EG Hernandez-Guzman G Bender CL Pierce NE Ausubel FM 2005 *Pseudomonas syringae* manipulates systemic plant defenses against pathogens and herbivores. Proceedings of the National Academy of Sciences, USA 102, 1791–1796 10.1073/pnas.0409450102 PMC547856 15657122

[CIT0009] De Vos M Van Oosten VR Van Poecke RMP 2005 Signal signature and transcriptome changes of Arabidopsis during pathogen and insect attack. Molecular Plant-Microbe Interactions 18, 923–937 16167763 10.1094/MPMI-18-0923

[CIT0010] Dicke M 1986 Volatile spider-mite pheromone and host-plant kairomone, involved in spaced-out gregariousness in the spider-mite *Tetranychus urticae* . Physiological Entomology 11, 251–262

[CIT0011] Dicke M Baldwin IT 2010 The evolutionary context for herbivore-induced plant volatiles: beyond the ‘cry for help’. Trends in Plant Science 15, 167–175 20047849 10.1016/j.tplants.2009.12.002

[CIT0012] Dicke M Gols R Ludeking D Posthumus MA 1999 Jasmonic acid and herbivory differentially induce carnivore-attracting plant volatiles in lima bean plants. Journal of Chemical Ecology 25, 1907–1922

[CIT0013] Dicke M van Loon JJA Soler R 2009 Chemical complexity of volatiles from plants induced by multiple attack. Nature Chemical Biology 5, 317–324 10.1038/nchembio.169 19377458

[CIT0014] Dicke M Vanbeek TA Posthumus MA Bendom N Vanbokhoven H Degroot AE 1990 Isolation and identification of volatile kairomone that affects acarine predator–prey interactions—involvement of host plant in its production. Journal of Chemical Ecology 16, 381–396 24263497 10.1007/BF01021772

[CIT0015] Engelberth J Koch T Schuler G Bachmann N Rechtenbach J Boland W 2001 Ion channel-forming alamethicin is a potent elicitor of volatile biosynthesis and tendril coiling. Cross talk between jasmonate and salicylate signaling in lima bean. Plant Physiology 125, 369–377 11154344 10.1104/pp.125.1.369 PMC61017

[CIT0016] Erb M Balmer D De Lange ES 2011 Synergies and trade-offs between insect and pathogen resistance in maize leaves and roots. Plant, Cell and Environment 34, 1088–1103 10.1111/j.1365-3040.2011.02307.x 21410707

[CIT0017] Gols R Roosjen M Dijkman H Dicke M 2003 Induction of direct and indirect plant responses by jasmonic acid, low spider mite densities, or a combination of jasmonic acid treatment and spider mite infestation. Journal of Chemical Ecology 29, 2651–2666 14969353 10.1023/b:joec.0000008010.40606.b0

[CIT0018] Halitschke R Stenberg JA Kessler D Kessler A Baldwin IT 2008 Shared signals—‘alarm calls’ from plants increase apparency to herbivores and their enemies in nature. Ecology Letters 11, 24–34 17961175 10.1111/j.1461-0248.2007.01123.x

[CIT0019] Harrison S Karban R 1986 Behavioral-response of spider-mites (*Tetranychus urticae*) to induced resistance of cotton plants. Ecological Entomology 11, 181–188

[CIT0020] Heil M 2008 Indirect defence via tritrophic interactions. New Phytologist 178, 41–61 18086230 10.1111/j.1469-8137.2007.02330.x

[CIT0021] Heil M Walters DR 2009 Ecological consequences of plant defence signalling. Plant Innate Immunity 51, 667–716

[CIT0022] Kessler A Baldwin IT 2001 Defensive function of herbivore-induced plant volatile emissions in nature. Science 291, 2141–2144 11251117 10.1126/science.291.5511.2141

[CIT0023] Kessler A Baldwin IT 2002 Plant responses to insect herbivory: The emerging molecular analysis. Annual Review of Plant Biology 53, 299–328 10.1146/annurev.arplant.53.100301.135207 12221978

[CIT0024] Koornneef A Leon-Reyes A Ritsema T Verhage A Den Otter FC Van Loon LC Pieterse CMJ 2008 Kinetics of salicylate-mediated suppression of jasmonate signaling reveal a role for redox modulation. Plant Physiology 147, 1358–1368 18539774 10.1104/pp.108.121392 PMC2442557

[CIT0025] Kunkel BN Brooks DM 2002 Cross talk between signaling pathways in pathogen defense. Current Opinion in Plant Biology 5, 325–331 12179966 10.1016/s1369-5266(02)00275-3

[CIT0026] Leon-Reyes A Du YJ Koornneef A Proietti S Korbes AP Memelink J Pieterse CMJ Ritsema T 2010 Ethylene signaling renders the jasmonate response of Arabidopsis insensitive to future suppression by salicylic acid. Molecular Plant-Microbe Interactions 23, 187–197 20064062 10.1094/MPMI-23-2-0187

[CIT0027] Leon-Reyes A Spoel SH De Lange ES Abe H Kobayashi M Tsuda S Millenaar FF Welschen RAM Ritsema T Pieterse CMJ 2009 Ethylene modulates the role of NONEXPRESSOR OF PATHOGENESIS-RELATED GENES1 in cross talk between salicylate and jasmonate signaling. Plant Physiology 149, 1797–1809 19176718 10.1104/pp.108.133926 PMC2663751

[CIT0028] Loivamaki M Mumm R Dicke M Schnitzler JP 2008 Isoprene interferes with the attraction of bodyguards by herbaceous plants. Proceedings of the National Academy of Sciences, USA 105, 17430–17435 10.1073/pnas.0804488105 PMC2582323 18987312

[CIT0029] Lou YG Du MH Turlings TCJ Cheng JA Shan WF 2005 Exogenous application of jasmonic acid induces volatile emissions in rice and enhances parasitism of *Nilaparvata lugens* eggs by the parasitoid *Anagrus nilaparvatae* . Journal of Chemical Ecology 31, 1985–2002 16132208 10.1007/s10886-005-6072-9

[CIT0030] Lu YB Liu SS Liu YQ Furlong MJ Zalucki MP 2004 Contrary effects of jasmonate treatment of two closely related plant species on attraction of and oviposition by a specialist herbivore. Ecology Letters 7, 337–345

[CIT0031] Mur LAJ Kenton P Atzorn R Miersch O Wasternack C 2006 The outcomes of concentration-specific interactions between salicylate and jasmonate signaling include synergy, antagonism, and oxidative stress leading to cell death. Plant Physiology 140, 249–262 16377744 10.1104/pp.105.072348 PMC1326048

[CIT0032] Ozawa R Arimura G Takabayashi J Shimoda T Nishioka T 2000 Involvement of jasmonate- and salicylate-related signaling pathways for the production of specific herbivore-induced volatiles in plants. Plant and Cell Physiology 41, 391–398 10845451 10.1093/pcp/41.4.391

[CIT0033] Pallini A Janssen A Sabelis MW 1997 Odour-mediated responses of phytophagous mites to conspecific and heterospecific competitors. Oecologia 110, 179–185 10.1007/s004420050147 28307422

[CIT0034] Pieterse CMJ Leon-Reyes A Van der Ent S Van Wees SCM 2009 Networking by small-molecule hormones in plant immunity. Nature Chemical Biology 5, 308–316 10.1038/nchembio.164 19377457

[CIT0035] Pieterse CMJ Van der Does D Zamioudis C Leon-Reyes A Van Wees SCM 2012 Hormonal modulation of plant immunity. Annual Review of Cell and Developmental Biology 28, 489–521 10.1146/annurev-cellbio-092910-154055 22559264

[CIT0036] Ponzio C Gols R Pieterse CMJ Dicke M 2013 Ecological and phytohormonal aspects of plant volatile emission in response to single and dual infestations with herbivores and phytopathogens. Functional Ecology 27, 587–598

[CIT0037] Rodriguez-Saona C Crafts-Brandner SJ Canas LA 2003 Volatile emissions triggered by multiple herbivore damage: Beet armyworm and whitefly feeding on cotton plants. Journal of Chemical Ecology 29, 2539–2550 14682532 10.1023/a:1026314102866

[CIT0038] Rodriguez-Saona CR Musser RO Vogel H Hum-Musser SM Thaler JS 2010 Molecular, biochemical, and organismal analyses of tomato plants simultaneously attacked by herbivores from two feeding guilds. Journal of Chemical Ecology 36, 1043–1057 20820890 10.1007/s10886-010-9854-7

[CIT0039] Schmelz EA Alborn HT Tumlinson JH 2003 Synergistic interactions between volicitin, jasmonic acid and ethylene mediate insect-induced volatile emission in *Zea mays* . Physiologia Plantarum 117, 403–412 12654041 10.1034/j.1399-3054.2003.00054.x

[CIT0040] Spoel SH Johnson JS Dong X 2007 Regulation of tradeoffs between plant defenses against pathogens with different lifestyles. Proceedings of the National Academy of Sciences, USA 104, 18842–18847 10.1073/pnas.0708139104 PMC2141864 17998535

[CIT0041] Stam JM Kroes A Li Y Gols R van Loon JJA Poelman EH Dicke M 2014 Plant interactions with multiple insect herbivores: from community to genes. Annual Review of Plant Biology 10.1146/annurev-arplant-050213-035937 (in press). 10.1146/annurev-arplant-050213-035937 24313843

[CIT0042] Stout MJ Thaler JS Thomma BPHJ 2006 Plant-mediated interactions between pathogenic microorganisms and herbivorous arthropods. Annual Review of Entomology 51, 663–689 10.1146/annurev.ento.51.110104.151117 16332227

[CIT0043] Thaler JS Agrawal AA Halitschke R 2010 Salicylate-mediated interactions between pathogens and herbivores. Ecology 91, 1075–1082 20462121 10.1890/08-2347.1

[CIT0044] Thaler JS Fidantsef AL Bostock RM 2002 Antagonism between jasmonate- and salicylate-mediated induced plant resistance: Effects of concentration and timing of elicitation on defense-related proteins, herbivore, and pathogen performance in tomato. Journal of Chemical Ecology 28, 1131–1159 12184393 10.1023/a:1016225515936

[CIT0045] Thaler JS Humphrey PT Whiteman NK 2012 Evolution of jasmonate and salicylate signal crosstalk. Trends in Plant Science 17, 260–270 22498450 10.1016/j.tplants.2012.02.010

[CIT0046] Thaler JS Stout MJ Karban R Duffey SS 2001 Jasmonate-mediated induced plant resistance affects a community of herbivores. Ecological Entomology 26, 312–324

[CIT0047] Turlings TCJ Loughrin JH Mccall PJ Rose USR Lewis WJ Tumlinson JH 1995 How caterpillar-damaged plants protect themselves by attracting parasitic wasps. Proceedings of the National Academy of Sciences, USA 92, 4169–4174 10.1073/pnas.92.10.4169 PMC41905 7753779

[CIT0048] van Dam NM Hadwich K Baldwin IT 2000 Induced responses in *Nicotiana attenuata* affect behavior and growth of the specialist herbivore *Manduca sexta* . Oecologia 122, 371–379 10.1007/s004420050043 28308288

[CIT0049] Voelckel C Baldwin IT 2004 Herbivore-induced plant vaccination. Part II. Array-studies reveal the transience of herbivore-specific transcriptional imprints and a distinct imprint from stress combinations. Plant Journal 38, 650–663 15125771 10.1111/j.1365-313X.2004.02077.x

[CIT0050] von Dahl CC Baldwin IT 2007 Deciphering the role of ethylene in plant–herbivore interactions. Journal of Plant Growth Regulation 26, 201–209

[CIT0051] Wei JN Wang LH Zhao JH Li CY Ge F Kang L 2011 Ecological trade-offs between jasmonic acid-dependent direct and indirect plant defences in tritrophic interactions. New Phytologist 189, 557–567 21039561 10.1111/j.1469-8137.2010.03491.x PMC3039750

[CIT0052] Wei JN Wang LZ Zhu JW Zhang SF Nandi OI Kang L 2007 Plants attract parasitic wasps to defend themselves against insect pests by releasing hexenol. PLoS ONE 2, e852 17786223 10.1371/journal.pone.0000852 PMC1955833

[CIT0053] Zarate SI Kempema LA Walling LL 2007 Silverleaf whitefly induces salicylic acid defenses and suppresses effectual jasmonic acid defenses. Plant Physiology 143, 866–875 17189328 10.1104/pp.106.090035 PMC1803729

[CIT0054] Zhang PJ Zheng SJ van Loon JJA Boland W David A Mumm R Dicke M 2009 Whiteflies interfere with indirect plant defense against spider mites in Lima bean. Proceedings of the National Academy of Sciences, USA 106, 21202–21207 10.1073/pnas.0907890106 PMC2795486 19965373

[CIT0055] Zheng SJ van Dijk JP Bruinsma M Dicke M 2007 Sensitivity and speed of induced defense of cabbage (*Brassica oleracea* L.): Dynamics of *BoLOX* expression patterns during insect and pathogen attack. Molecular Plant-Microbe Interactions 20, 1332–1345 17977145 10.1094/MPMI-20-11-1332

