# Supplementary material for: Reciprocal crosstalk between jasmonate and salicylate defence-signalling pathways modulates plant volatile emission and herbivore host-selection behaviour
Source: J Exp Bot. 2014 Apr 23;65(12):3289–98. doi: 10.1093/jxb/eru181 (PMC4071845; doi:10.1093/jxb/eru181)
Supplement: Supplementary Data [file supp_65_12_3289__index.html]

Reciprocal crosstalk between jasmonate and salicylate defence-signalling pathways modulates plant volatile emission and herbivore host-selection behaviour — Reciprocal crosstalk between jasmonate and salicylate defence-signalling pathways modulates plant volatile emission and herbivore host-selection behaviour — Reciprocal crosstalk between jasmonate and salicylate defence-signalling pathways modulates plant volatile emission and herbivore host-selection behaviour — Supplementary Data 

# Reciprocal crosstalk between jasmonate and salicylate defence-signalling pathways modulates plant volatile emission and herbivore host-selection behaviour

## Supplementary Data

Data files

**Files in this Data Supplement:**

- Supplementary Data - Supplementary Data
