# Supplementary material for: Reciprocal crosstalk between jasmonate and salicylate defence-signalling pathways modulates plant volatile emission and herbivore host-selection behaviour
Source: J Exp Bot. 2014 Apr 23;65(12):3289–98. doi: 10.1093/jxb/eru181 (PMC4071845; doi:10.1093/jxb/eru181)
Supplement: Supplementary Data [file supp_eru181_jexbot122853_file001.pdf]

Journal of Experimental Botany Supplementary data

Reciprocal crosstalk between jasmonate and salicylate defence-signalling pathways modulates plant volatile emission and herbivore host-selection behaviour

Jianing Wei [1, 2], Joop J. A. van Loon [1], Rieta Gols[1], Tila R. Menzel[1], Na Li[2], Le Kang [2], and Marcel Dicke [1, *]

[1] Laboratory of Entomology, Wageningen University, P.O. Box 8031, 6700 EH, Wageningen, The Netherlands;

[2] State Key Laboratory of Integrated Management of Pest Insects and Rodents, Institute of Zoology, Chinese Academy of Sciences, Beijing 100080, P. R. China. weijn@ioz.ac.cn; joop.vanloon@wur.nl; Rieta.Gols@wur.nl; tila.menzel@wur.nl; lina5sxnd@sina.com; lkang@ioz.ac.cn; marcel.dicke@wur.nl.

[*] To whom correspondence should be addressed: Marcel Dicke, E-mail: marcel.dicke@wur.nl.

This file contains information on methods and supplementary Figures S1-S8 with Legends and Supplementary Tables S1-S5.

Supplementary methods

Headspace volatile collection

Volatiles emitted by Lima bean plants were collected with a dynamic headspace collection system using a pump (Loivamaki *et al.*, 2008). Briefly, the plant's pot was removed and the roots and soil were packed in aluminum foil. Then it was transferred to a 2.5-L glass jar. Incoming air was filtered by passing through a tube filled with 120 mg Tenax TA (Grace-Alltech). The system was purged at a flow rate of 2.5 L/min for 1 h with filtered air before trapping volatiles onto the adsorbents. After that, air was sucked out of the jar at a flow rate of 100 mL/min by passing through a tube filled with 120 mg Tenax TA. Headspace volatiles from plants exposed to different treatments were collected for a period of 2 h between 12:00 AM and 2:00 PM. Fresh weight of the plant (shoot) was determined immediately after the experiments. Headspace collections were carried out in a growth chamber at 23 ± 1 °C, 60 ± 5% R.H, L8:D16 photoperiod, and 90–110 μmol photons m^{-2} s^{-1} PPFD at canopy height.

An SPME fiber (PDMS/DVB 65μm) was used to adsorb the volatiles from a leaf disk, which was taken from a leaf of a bean plant (*Phaseolus vulgaris)* and put in a glass vial (Waters, 20ml, 27.5mm×57mm), within 15 min.

Chemical analysis of headspace volatiles

Headspace samples were analyzed as described in detail by Zhang et al. (2009). Briefly, volatile traps were flushed with helium (30 mL/min) for 20 min to remove moisture and oxygen. Then, samples were thermally desorbed at 220 °C for 5 min

and refocused on a sorbent trap at 0 °C. Volatiles were injected on the analytical column (Rtx-5ms, 30 m × 0.25 mm ID, 1.0-μm film thickness, Restek) with a split flow of 20 mL/min by heating the cold trap to 250 °C for 3 min. The temperature program of the GC was as follows: 40 °C (3-min hold), 10 °C/min to 280 °C (3-min hold). The column effluent was ionized by electron impact ionization (70 eV). Mass scanning was done from 33 to 250 *m*/*z*. Compounds were identified by comparing the mass spectra with those of authentic standards (Table S1) or with NIST02, NIST05, Wiley libraries and the Wageningen Mass Spectral Database of Natural Products. Quantification of identified compounds was based on comparison with a set of authentic compounds ((*Z*)-3-hexen-ol, (*Z*)-3-hexenyl acetate, (*E*)-β-ocimene, β-caryophyllene, methyl salicylate) injected in different concentrations ranging from 100 ng to 10 μg/μL in hexane. Response factors were linear for all reference compounds within this concentration range (Zhang *et al.*, 2009).

For chemical analysis of SPME samples, an Agilent gas chromatograph (GC) system (6890N) coupled with a mass spectrometer (MS) (5973 MSD, Agilent Technologies, Inc., USA) equipped with an HP5-MS column (30 m × 0.25 mm ID, 0.25 μm film thickness) was used. The GC oven temperature was kept at 40°C for 1 min and then increased to 160°C at a rate of 10°C min^{-1}, followed by a rate of 20°C min^{-1} to 280°C. The GC-MS electron impact source was operated in the scan mode with the MS source temperature at 230°C and MS Quad at 150 °C. Volatile compounds were identified by comparing their retention time and spectra with synthetic standards (Zhang *et al.*, 2009).

Quantitative real-time PCR

Total RNA extraction and purification were done as described in the handbook of RNeasy Plant Mini Kit (Qiagen Group, Valencia, CA, USA). cDNA synthesis was performed as reported by Zheng et al. (2007). To quantify *lipoxgenase* (*LOX*), *P. lunatus Ocimene Synthase* (*PlOS*), and *pathogenesis-related protein* (*PR-2* (β-1,3-glucanase)) transcript levels, real-time quantitative RT-PCR was performed in a Rotor-Gene 6000 machine (Corbett Research) with a 72-well rotor (Zhang *et al.*, 2009). Briefly, the amplification reactions were performed in 25 μL final volume containing 12.5 μL ABsolute TM QPCR SYBR Green Mix (ABgene), 1 μL forward primer (2 μM) and reverse primer (2 μM) pairs (final primer concentration: 80 nM), and 1μL cDNA (10 ng/μL) first strand template. The PCR program was the same as described by Zheng et al. (2007). The gene-specific primers of *LOX* (GenBank accession X63521), *PlOS* (GenBank accession EU194553), *PR-2* (GenBank accession M13968) and *PlACT1* (GenBank accession DQ159907) as housekeeping gene were designed with the Beacon Designer software (Premier Biosoft International) set to an annealing temperature of 56 °C. *LOX* primers were F-*LOX* (5′-GGAATGGGACAGGGTTTATG-3′) and R-*LOX* (5′- CAAAGTCACTGGGCTTCTCA- 3′). *PlOS* primers were F-*PlOS* (5′-TGCATGGGTCTCAGTCTCTG-3′) and R- *PlOS* (5′-TGCTGCTTCCCCTCTCTCTA-3′). *PR-2* primers were F-*PR-2* (5′- GTGGATGCT GTTGTTGGTTG -3′) and R- *PR-2* (5′- GTCGAAGGTGGACCTGGATA -3′). *PlACT1* primers were F-*PlACT1* (5′- CCAAGGCTAACCGTGAAAAG-3′) and R-*PlACT1* (5′-AGCCAGATCAAGACGAAGGA-3′). The *LOX*, *PR-2*, *PlOS* expression relative to

PlACT1 expressions were quantified by the $2^{-\Delta\Delta CT}$ method (Real-Time PCR Application Guide, Bio-Rad).

Statistical Analysis

We used the PLS-DA (Projection to Latent Structures Discriminant Analysis) extension of the SIMCA P+ 12.0 software program, (Umetrics AB, Umeå, Sweden). This projection method separates groups of observations that are assigned to specific classes (here treatments) by rotating the principle component or PC's such that a maximum separation among classes is obtained (Eriksson et al. 2006). To achieve this, a Y-data matrix of dummy variables is included, which assigns a sample to its respective class. This analysis approximates the point 'swarm' in X (matrix with volatile compounds) and Y in PLS components in such a way that maximum covariation between the components in X and Y is achieved. The program's cross validation procedure evaluates the significance of each additional component (starting with none) by comparing the goodness of fit (R2) and the predictive value (Q2) of the extended model with that of the reduced model. Per sample, amounts of the individual volatile compounds were measured as ng produced per g fresh mass of shoot tissue emitted per h. Data mean-centered, and scaled to unit variance before they were subjected to the analysis.

References

Loivamaki M, Mumm R, Dicke M, Schnitzler JP. 2008. Isoprene interferes with the

attraction of bodyguards by herbaceous plants. *Proceedings of the National Academy of Sciences, USA* **105**, 17430-17435.

Zhang PJ, Zheng SJ, van Loon JJA, Boland W, David A, Mumm R, Dicke M. 2009. Whiteflies interfere with indirect plant defense against spider mites in Lima bean. *Proceedings of the National Academy of Sciences, USA* **106**, 21202-21207.

Eriksson L, Johansson E, Kettaneh-Wold N, Wikström JTC, Wold S. 2006. Multi- and Megavariate Data Analysis. Part 1: Basic Principles and Applications. Umetrics AB, Umeå, Sweden.

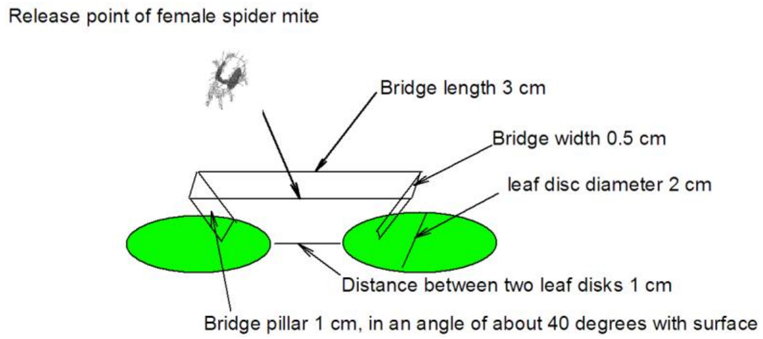

Fig. S1. Two-choice set-up to investigate odour-based attraction of female spider mites to leaf disks exposed to phytohormone treatments. The bridge was cut from transparent acetate sheet and folded to a trapezoid shape. This setup was modified after Van den Boom et al. (2004) and Grostal and Dicke (1999, 2000).

References

Van den Boom CEM, Van Beek TA, Posthumus MA, De Groot A, Dicke M. 2004. Qualitative and quantitative variation among volatile profiles induced by *Tetranychus urticae* feeding on plants from various families. *Journal of Chemical Ecology* **30**, 69-89.

Grostal P, Dicke M. 1999. Direct and indirect cues of predation risk influence behaviour and reproduction of prey: a case for acarine interactions. *Behavioral Ecology* **10**, 422-427.

Grostal P, Dicke M. 2000. Recognising one’s enemies: a functional approach to risk assessment by prey. *Behavioral Ecology and Sociobiology* **47**, 258-264.

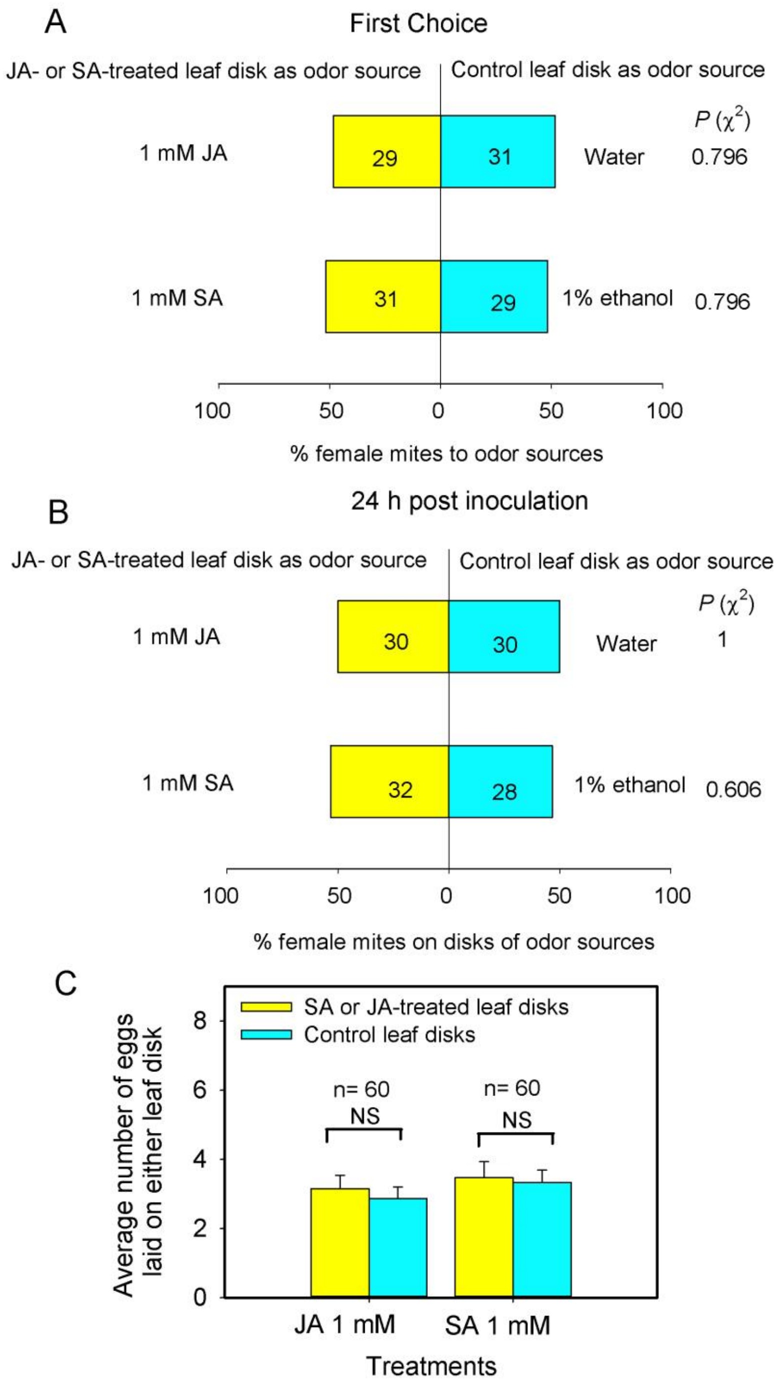

Fig. S2. Short-term effect of JA or SA treatments on preference of female spider mites. The first choice (A), choice at 24 h post inoculation (B), and average numbers of eggs laid (C) by female spider mites on JA or SA-treated Lima bean leaf disks were compared with that on control leaf disks. Water, a solution of 1% ethanol in water (controls for JA- and SA-application respectively), and solutions of 1mM JA

or SA were sprayed on Lima bean leaves and the treated plants were incubated for 20 min. After that, the phytohormone-treated leaf disks and control leaf disks were used as odour sources to determine if JA or SA itself have direct effects on preference of spider-mites compared with control through volatilization. The behavioural assays were done within 1 h since phytohormone application. These experiments were repeated 3 times at three different days and a total of 60 mites were individually used in each treatment. χ^2 test for significant differences between numbers of mites in each disk. In panel C: NS: not significant. 1 mM JA versus control: t = 0.211, df = 59, P = 0.834; 1 mM SA versus control: t = 0.812, df = 59, P = 0.420 (two-tailed paired t-test). Before parametric analysis oviposition data were log (x+1) transformed to correct for heterogeneity of variances.

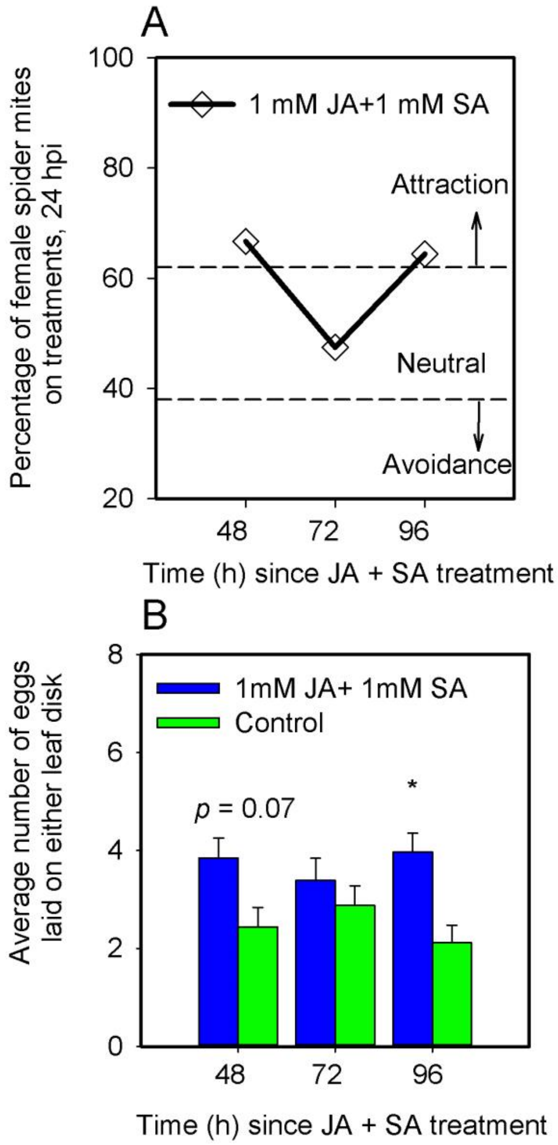

Fig. S3. Time course of effects of JA + SA-treatment versus control (1% ethanol) on distribution and oviposition of female spider mites on Lima bean leaf disks. (A), percentage of mites observed on JA + SA-treated leaf disks 24 hpi. (B), numbers of eggs laid by female spider mites on JA+SA-treated Lima bean disk versus that on control leaf disk (mean ± SE). These experiments were repeated 3 times at three different days. In total 60 mites were studied for each treatment and time point (n = 60). In (A), data points located in areas above or below dashed lines indicated by arrows designated 'attraction' or 'avoidance' indicate a choice distribution

significantly different from 50:50 (χ^2 test) (see Table S1 for details on statistical analysis). In (B), significance of differences between average number of eggs laid on either treatment or control leaf disks at each time point were analysed using the paired (two tailed) *t*-test; * $P < 0.05$, ** $P < 0.01$, *** $P < 0.001$ ((see Table S2 for details on statistical analysis)). Before parametric analysis, oviposition data were log (x+1) transformed to correct for heterogeneity of variances.

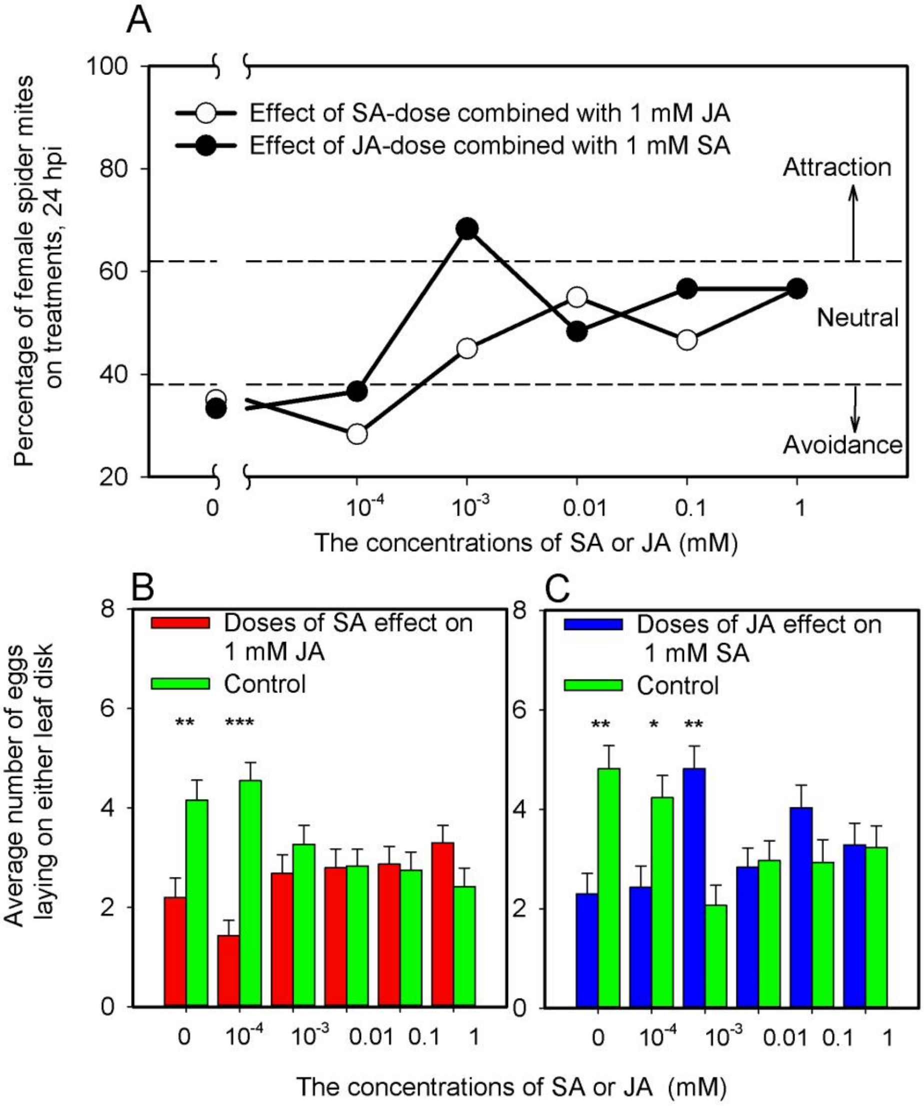

Fig. S4. Distribution and oviposition of female spider mites on Lima bean leaf disks in a two-choice situation as affected by combined application of JA (1 mM) and SA at 5 different concentrations (open symbols) and SA (1 mM) and JA at 5 different concentrations (filled symbols) *vs.* control disks. These experiments were repeated 3 times at three different days. In total 60 mites were individually studied for each treatment and time point (n = 60). In (A), data points located in areas above or below dashed lines indicated by arrows designated 'attraction' or 'avoidance' indicate a choice distribution significantly different from 50:50 (χ^2 test) (see Table S1 for

details on statistical analyses). In (B) and (C), paired (two tailed) *t*-test for significant differences of average number of eggs laid on treatment and control leaf disks at each time point, * $P<0.05$, ** $P<0.01$ (see Table S2 for details on statistical analyses). Before parametric analysis oviposition data were log (x+1) transformed to correct for heterogeneity of variances.

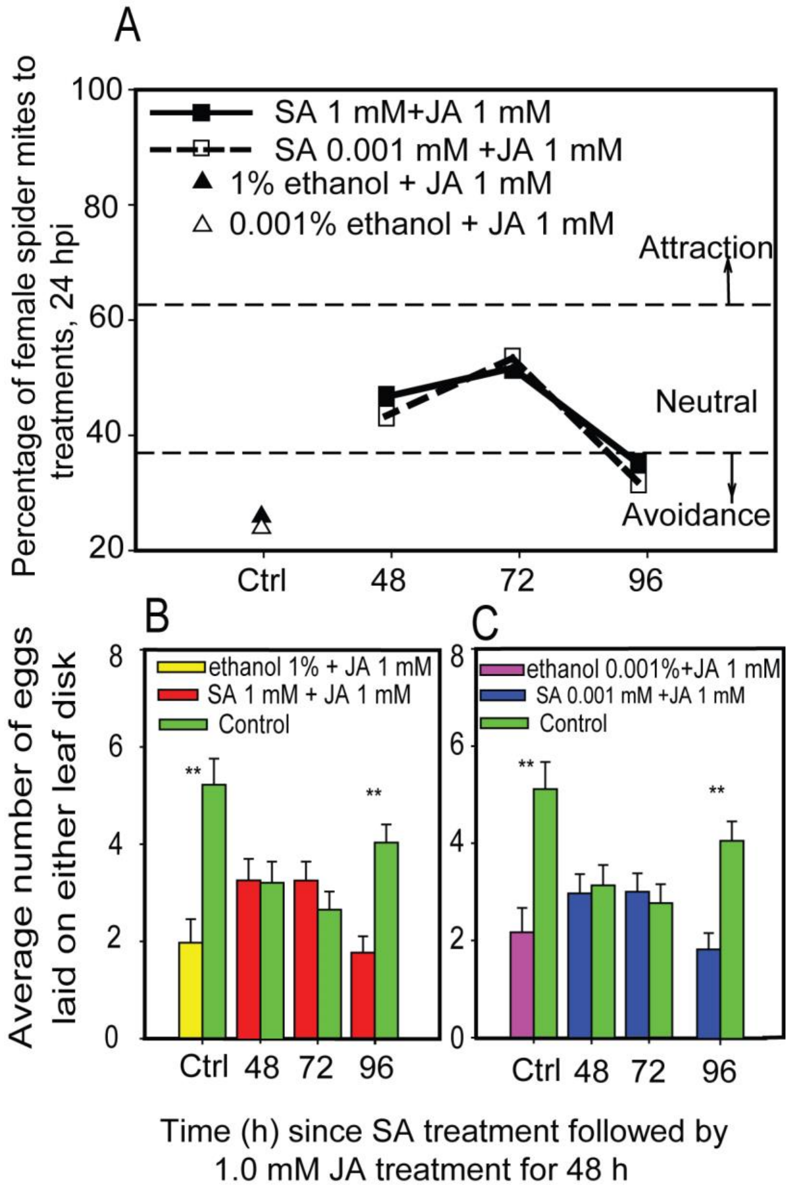

Fig. S5. Persistence of the SA-mediated antagonistic effect on JA-induced repellency to spider mites. These experiments were repeated 3 times at three different days. In total 60 mites were individually studied for each treatment and time point (n =60). In (A), data points located in areas above or below dashed lines indicated by arrows designated 'attraction' or 'avoidance' indicate a choice distribution significantly different from 50:50 (χ^2 test) (see Table S1 for details on statistical analyses). Ctrl 48 represents treatment that the plants were sprayed with

the solutions of either 0.001% or 1% ethanol in the negative control experiments. In (B) and (C), paired (two tailed) *t*-test for significant differences of average number of eggs laid on treatment and control leaf disks at each time point, * $P<0.05$, ** $P<0.01$ (see Table S2 for details on statistical analyses). Before parametric analysis oviposition data were log (x+1) transformed to correct for heterogeneity of variances.

Volatile analysis of leaf disks cut either from healthy bean plants or from bean plants treated with 1 mM JA for 24h, or with 1mM SA for 48h.

GC-MS analysis shows that green leaf volatiles (GLVs), such as (*Z*)-3-hexenal, (*E*)-2-hexenal, (*Z*)-3-hexen-ol, 1-octen-3-ol, and (*Z*)-3-hexenyl acetate, are released immediately from freshly dissected leaf disks and dominate the volatile blends (Fig. S6). However, the amount of these volatiles decreased significantly by ca. 5-50-fold within 10 min since the leaf disk had been prepared (Fig. S6). A comparison between GLV emissions from leaf disks cut from control untreated plants and from plants treated with 1 mM JA for 24 h showed that the amount of (*Z*)-3-hexen-ol (#3) was released in significantly higher amounts from leaf disks from control plants than from leaf disks from JA-treated plants, whereas the amount of (*Z*)-3-hexenyl acetate (#5) is lower than that recorded for leaf disks from JA-treated plants. In addition, JA-induced compounds (#6 to #12), such as (*E*)-β-ocimene, (3*E*)-4,8-dimethyl-1,3,7-nonatriene (DMNT), (*Z*)-3-hexenyl propionate, (*Z*)-3-hexenyl butyrate, and caryophyllene, were undetectable in leaf disks from healthy plants (Fig. S7A). A comparison of GLVs emitted by leaf disks from healthy and SA-treated plants shows that the amounts of (*Z*)-3-hexen-ol and (*Z*)-3-hexenyl acetate are higher in disks from SA-treated plants, but the amount of (*Z*)-3-hexenal is higher in disks from healthy plants. Similarly, SA-induced compounds (#6 to #9) were undetectable in leaf disks from control plants (Fig. S7B). Therefore, the transient reactions of healthy, JA-treated, and SA-treated plants to the treatment of punching the leaf disks are transient release of GLVs, but this treatment did not result in JA or SA-induced volatile compounds (Fig. S6 and S7) in healthy leaf disks, supporting

the conclusion that the mite choices in our studies are due to the treatments of JA and SA, not to punching the leaf disks, which was done for both treatment and control alike.

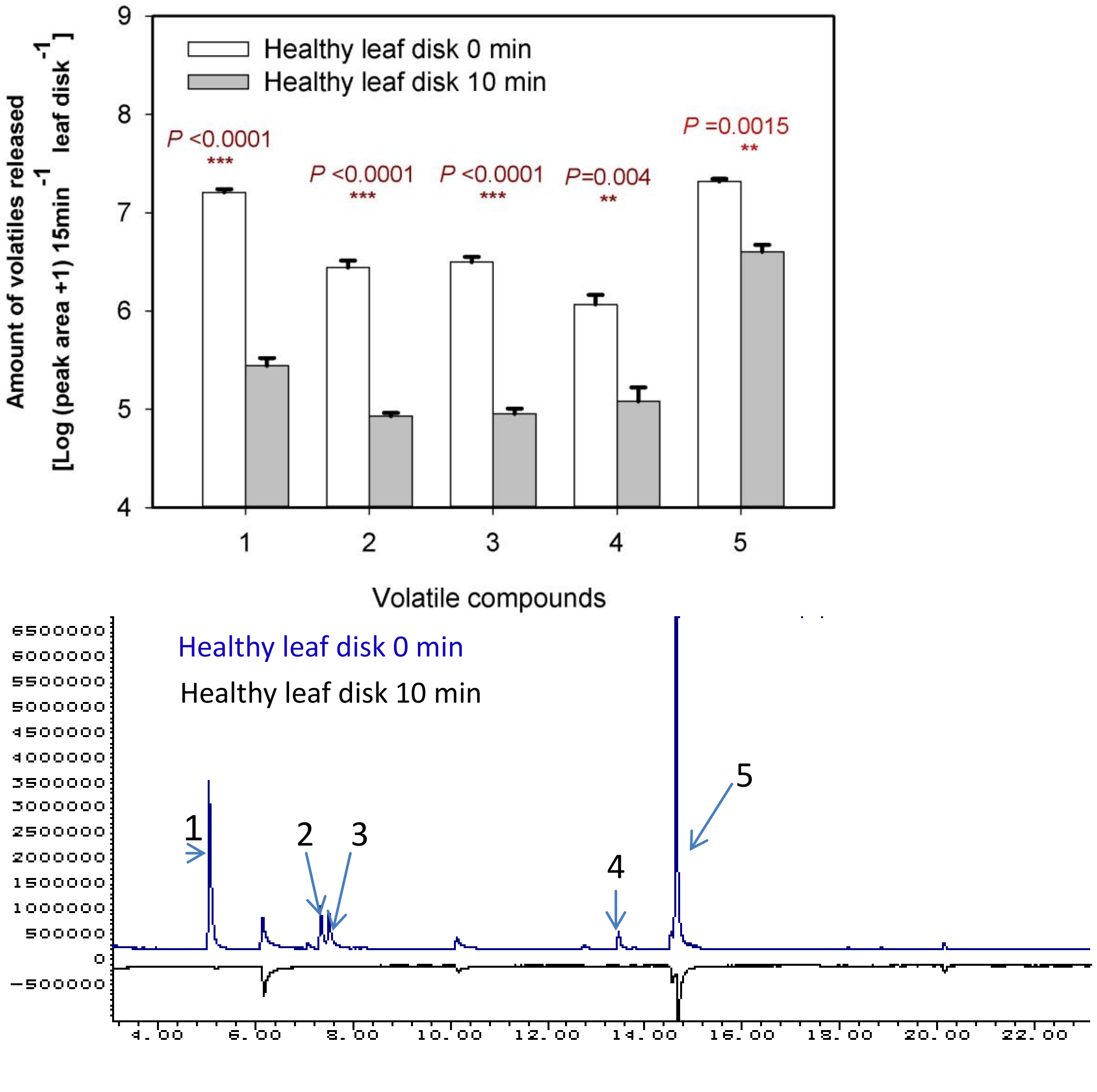

Fig. S6. Green leaf volatiles collected from the headspace of leaf disks taken from healthy bean plants in a glass vial. Healthy leaf disk 0 min represents a leaf disk that was sealed in a glass vial immediately after dissecting and its volatiles were adsorbed by SPME for 15 min; Healthy leaf disk 10 min denotes a leaf disk that was first exposed to air for ten minutes after dissection, and then placed in a glass vial for

collection of headspace for 15 min. The TICs (Total ion chromatograms) of volatile profiles of healthy leaf disk 0 min (blue trace) and an untreated control leaf disk 10min (black trace). Volatile compounds, 1. (*Z*)-3-hexenal; 2. (*E*)-2-hexenal; 3. (*Z*)-3-hexen-ol. 4. 1-octen-3-ol; 5. (*Z*)-3-hexenyl acetate. The release rate of compounds is represented by the transformation of the peak area by Log (X+1). Data within a compound are analyzed with a paired two-tailed t-test, ** $P<0.01$, *** $P< 0.001$. These experiments were repeated 3 times.

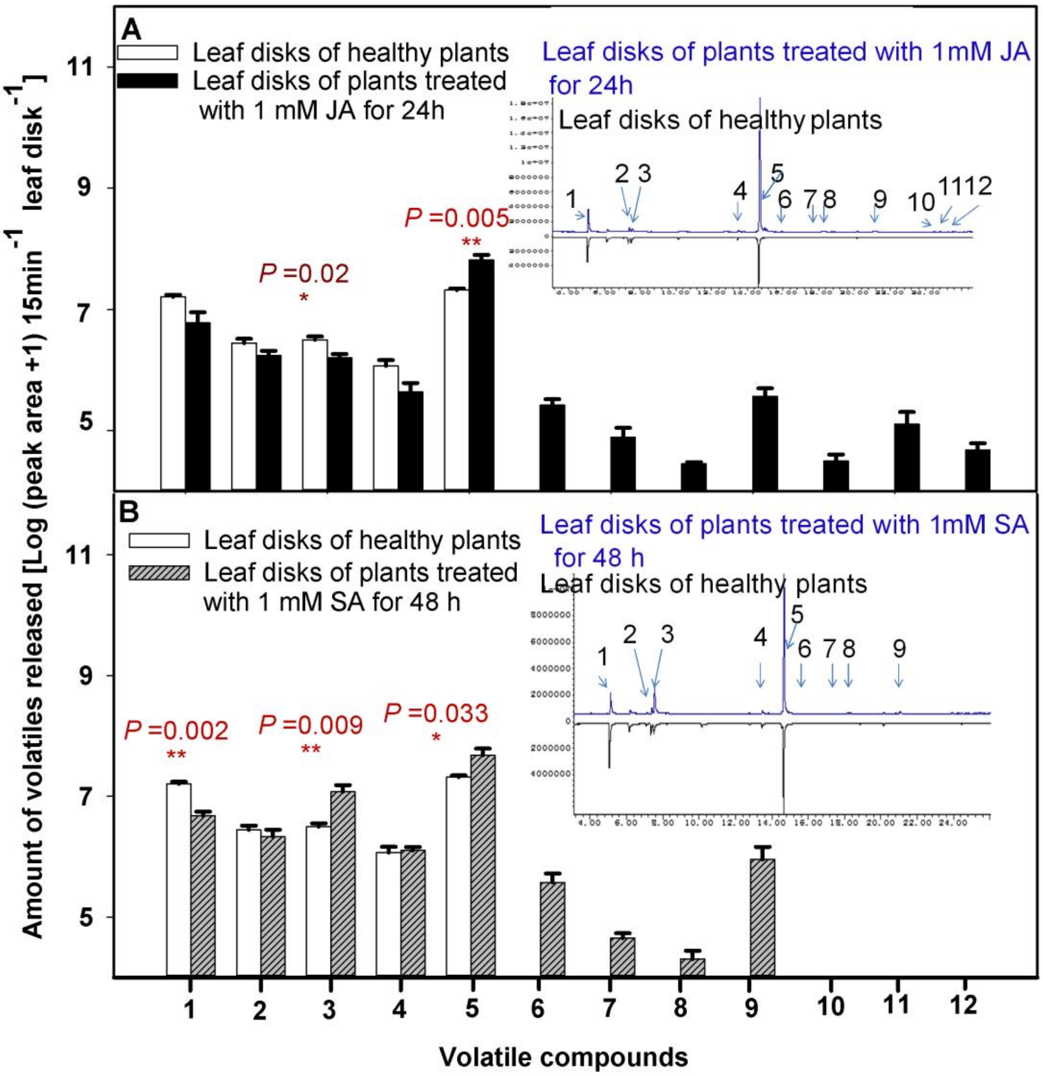

Fig. S7. Volatile compounds released from leaf disks taken from healthy bean plant, plants treated with 1 mM JA for 24h (A), and plants treated with 1 mM SA for 48h (B) immediately after dissecting and was absorbed by SPME for 15 min in a glass vial. In panel (A), the TICs of healthy leaf disk (black trace) and 1 mM JA-treated leaf disk (blue trace). In panel (B), the TICs of healthy leaf disk (black trace) and 1 mM SA-treated leaf disk (blue trace). Volatile compounds, 1. (*Z*)-3-hexenal; 2. (*E*)-2-hexenal; 3. (*Z*)-3-hexen-ol. 4. 1-octen-3-ol; 5. (*Z*)-3-hexenyl acetate; 6.

(*E*)-β-ocimene; 7. (*Z*)-3-hexenyl propionate; 8. (3*E*)-4,8-dimethyl-1,3,7-nonatriene (DMNT); 9. (*Z*)-3-hexenyl butyrate; 10. α-copaene; 11. Caryophyllene; 12. α–humulene. The release rate of compounds is represented by the transformation of the peak area by Log (X+1). Data within a compound are analyzed with a paired two-tailed t-test, ** $P<0.01$, *** $P< 0.001$. These experiments were repeated 3 times.

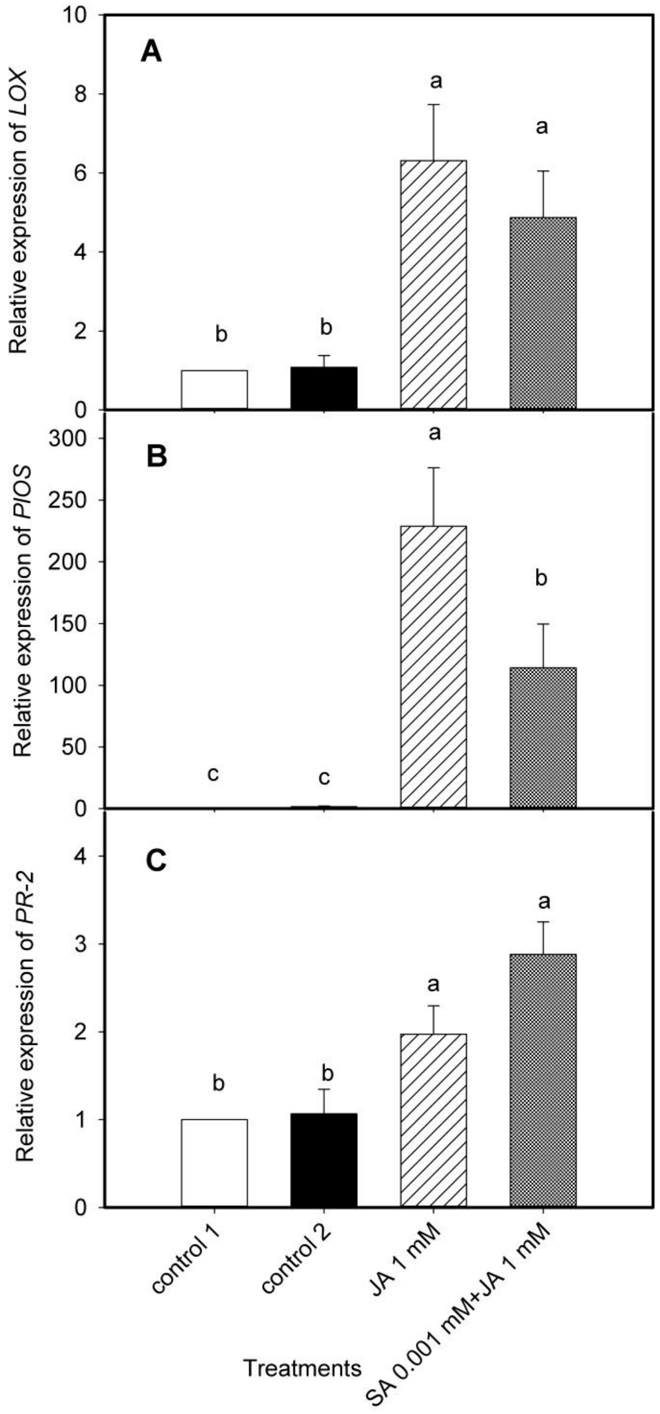

Fig. S8. Transcript levels of *LOX* (A), *PlOS* (B), and *PR-2* (C) relative to control (water treated) plants in Lima bean plants sprayed with 1mM JA, or 0.001mM SA plus 1 mM JA. Transcript levels of the three genes in plants treated with water (control 1), 0.001% ethanol 24h plus water for 24h (Control 2), 1 mM JA for 24 h

only, or plants treated with 0.001 mM SA for 24 h and then with 1 mM JA and incubated for another 24 h before RNA extraction. *LOX*, *PlOS*, and *PR-2* transcript levels have been normalized to the amount of *PlACT1* transcripts in each sample. Values are the mean (±SE) of three biological replicates. Different letters above bars indicate significant differences in the transcript levels among treatments (analysis of variance (ANOVA) and Tukey's honestly significant difference (HSD) test $P < 0.05$).

Table S1. Statistical analyses of odour-based preferences of female spider mites to leaf disks of different treatments in all experiments

Experiments	Preferences and times or doses *	N Number of mites to treatment vs control †			χ^2 -value	*P*-value ‡
		Rep1	Rep2	Rep3		
	First choice					
	24 h	5:15	7:13	6:14	9.6	0.00195**
	48 h	8:12	10:10	13:7	0.267	0.605
	72 h	14:6	15:5	12:8	8.067	0.00451**
	96 h	11:9	9:11	9:11	0.067	0.796
1mM JA versus water control	120 h	13:7	10:10	11:9	1.067	0.302
	Distribution 24 h post inoculation (hpi)					
	48 h	7:13	4:16	7:13	11.267	0.00079***
	72 h	10:10	12:8	13:7	3.26	0.071
	96 h	13:6	14:6	15:5	9.6	0.00195**
	120 h	9:11	6:14	11:9	1.067	0.302
	144h	8:12	10:10	11:8	0	1
	First choice					
1mM SA	24 h	10:10	11:9	9:11	0	1
versus	48 h	7:13	8:12	5:15	9.6	0.00195**

1% ethanol	72 h	13:7	15:5	15:5	11.267	0.00079***
	96 h	11:9	9:11	12:8	0.267	0.606
	120 h	10:10	11:9	9:11	0	1
	Distribution 24 hpi					
	48 h	11:9	10:10	8:12	0.067	0. 796
	72 h	8:12	7:13	7:13	4.267	0.0389*
	96 h	14:6	17:3	13:7	13.067	0.0003***
	120 h	10:10	11:9	11:9	0.267	0.606
	144h	10:10	10:10	8:12	0.267	0.606
Combined 1 mM JA and 1 mM SA versus control	First choice					
	24 h	10:10	10:10	9:11	0.067	0.796
	48 h	11:9	11:9	10:10	0.267	0.606
	72 h	12:8	13:7	14:6	5.40	0.0201*
	Distribution 24 hpi					
	48 h	11:8	7:13	14:6	4.898	0. 0269*
	72 h	11:8	7:12	10:10	0.153	0.696
	96 h	14:6	12:8	14:6	6.667	0.0098**
SA-dose on JA-SA cross-talk	First choice					
	0 mM	7:13	6:14	7:13	9.6	0.0023**
	0.0001 mM	6:14	6:14	7:13	8.067	0.00451**
	0.001mM	11:9	11:9	10:10	0.267	0.605

	0.01 mM	10:10	10:10	10:10	0	1
	0.1 mM	12:8	10:10	11:9	0.60	0.439
	1 mM	11:9	9:11	10:10	0	1
	Distribution 24 hpi					
	0 mM	7:13	7:13	7:13	5.4	0.0148*
	0.0001 mM	6:14	6:14	5:15	11.267	0.0008***
	0.001mM	9:11	10:10	8:12	0.6	0.439
	0.01 mM	14:6	7:13	12:8	0.60	0.439
	0.1 mM	9:11	9:11	10:10	0.267	0.606
	1 mM	12:8	10:10	12:8	1.067	0.302
	First choice					
	0 mM	7:13	7:13	6:14	6.67	0.00982**
	0.0001 mM	7:13	7:13	7:13	5.4	0.020*
	0.001mM	13:7	12:8	13:7	4.267	0.0389*
JA-dose on	0.01 mM	9:11	12:8	9:11	0	1
JA-SA	0.1 mM	11:9	9:11	10:10	0	1
cross-talk	1 mM	11:9	11:9	11:9	0.60	0.439
	Distribution 24 hpi					
	0 mM	8:12	6:14	6:14	6.67	0.00982**
	0.0001 mM	8:12	7:13	7:13	4.267	0.039*
	0.001mM	15:5	12:8	14:6	8.067	0.00451**

	0.01 mM	10:10	12:8	7:13	0.067	0.796
	0.1 mM	10:10	9:11	10:10	0.067	0.796
	1 mM	10:10	12:8	12:8	0.067	0.796
Persistence of 1 mM SA-mediated antagonism to 1mM JA treatment	First choice					
	Ctrl 24 h+24 h	7:13	6:14	7:13	6.67	0.0098**
	SA72h+JA 24 h	7:13	7:13	5:15	8.067	0.0045**
	SA48h+JA 24 h	13:7	13:7	11:9	3.267	0.071
	SA24 h+JA24 h	11:9	9:11	10:10	0	1
	Distribution 24 hpi					
	Ctrl 48 h+48 h	4:16	7:13	4:16	15	0.00011***
	SA96h+JA48 h	7:13	8:12	6:14	4.267	0.0389*
	SA72h+JA48 h	10:10	12:8	10:10	0.267	0.606
	SA 48h+JA48 h	10:10	9:11	10:10	0.067	0.796
Persistence of 0.001 mM SA-mediated antagonism to 1mM JA treatment	First choice					
	Ctrl 24 h+24 h	6:14	6:14	5:15	11.267	0.00079***
	SA 72h+JA24h	6:14	7:13	7:13	6.67	0.0098**
	SA 48h+JA 24h	10:10	10:10	12:8	0.267	0.606
	SA24 h+JA 24h	12:8	11:9	10:10	0.60	0.439
	Distribution 24 hpi					
	Ctrl 48 h+48 h	6:14	6:14	4:16	13.07	0.00030***
	SA 96h+JA48 h	7:13	7:13	6:14	6.67	0.0098**

SA 72h+JA48 h	11:9	10:10	12:8	0.60	0.439
SA48h+JA 48h	11:9	9:11	7:13	0.60	0.439

[*] The χ^2 test for significance of differences between proportions was used to analyse mite preference behaviour.

[†] "N treatment vs control" are the numbers of spider mites choosing treatment versus control presented separately for each replicate day. Rep1 is the data of replicate 1 and Rep2 and Rep3 are of replicates 2 and 3 respectively.

[‡] : * $P < 0.05$; ** $P < 0.01$; *** $P < 0.001$. Red asterisks denote significance of repellency and blue asterisks represent significance of attractiveness. No asterisks means neutrality.

Table S 2. Statistical analyses of numbers of eggs laid by female spider mites on JA-or/ and SA-treated Lima bean disks versus that on control leaf disks

Experiments	Times or doses [*]	*df*	*t* -value	*P*-value
	48 h	59	-3.314	0.00157**
1 mM JA	72 h	59	0.682	0.497
versus	96 h	59	2.634	0.010 *
water control	120 h	59	- 1.316	0.193
	144h	59	- 0.217	0.829
	48 h	59	0.554	0.582
1 mM SA	72 h	59	- 2.543	0.014*
versus	96 h	59	4.26	0.00007***
1% ethanol	120 h	59	1.723	0.101
	144h	59	- 0.812	0.419
Combined 1 mM JA and 1 mM SA versus control	48 h	59	1.82	0.07
	72 h	58	0.339	0.735
	96 h	58	2.459	0.017*
	0 mM	59	- 2.489	0.0156*
SA-dose on	0.0001 mM	59	- 4.632	0.00002***
JA-SA	0.001 mM	59	- 0.745	0.457
cross-talk	0.01 mM	59	0.284	0.813
	0.1 mM	59	0.339	0.735

	1 mM	59	1.473	0.146
JA-dose on JA-SA cross-talk	0 mM	59	- 2.735	0.0082**
	0.0001 mM	59	2.846	0.007**
	0.001mM	59	3.292	0.0017**
	0.01 mM	59	- 0.097	0.922
	0.1 mM	59	- 0.632	0.547
	1 mM	59	1.299	0.198
Persistence of 1 mM SA-mediated antagonism to 1mM JA treatment	Ctrl 48 h+48 h	59	- 3.314	0.00157**
	SA 96h + JA 48 h	59	- 3.256	0.00187**
	SA 72h + JA 48 h	59	0.779	0.438
	SA 48 h +JA 48 h	59	- 0.779	0.438
Persistence of 0.001 mM SA-mediated antagonism to 1mM JA treatment	Ctrl 48 h+48 h	59	- 3.202	0.00232**
	SA 96h + JA 48 h	59	-2.861	0.0053**
	SA 72h + JA 48 h	59	0.364	0.717
	SA 48 h +JA 48 h	59	- 0.190	0.849

* A paired two-tailed *t*-test was employed to assess the significance of differences in average number of eggs deposited on treatment and control leaf disks at each time point or dosage. Before parametric analysis, oviposition data were log (x+1)

transformed to correct for heterogeneity of variances.

[†] * $P < 0.05$; ** $P < 0.01$; *** $P < 0.001$.

Table S3 Absolute and relative (%) amount of volatiles released from Lima bean plants treated with 0.001mM SA for 24h and then with 1.0 mM JA application for another 24h and from plants with control (0.001% ethanol treatment) and 1mM JA applications in same time interval.

	**Chemical compound**	Retention Time	M/Z ‡ fragments	0.001% ethanol+1mM JA		0.001mMSA+1mM JA	
				Quantities (ng / g / h)	Relative %	Quantities (ng / g / h)	Relative %
	Major compounds						
1	*(Z)*-3-Hexen-ol*	10.06	67	8.90±1.71	0.48±0.06	3.94± 0.99	0.37 ±0.10
2	(*Z*)-3-Hexenyl acetate *	12.95	43	363.34±99.75	18.62±3.61	158.72± 17.18	17.86± 2.82
3	(*Z*)-β-ocimene*	13.55	93	61.73±3.36	3.68±0.25	44.59± 7.44	4.04 ±0.24
4	(*E*)-β-ocimene*	13.77	93	961.47±36.63	57.73±3.87	552.99± 102.68	48.61± 4.22
5	Linalool*	14.65	93	81.93±9.51	4.82±0.57	61.31± 13.56	6.21 ±1.21
6	DMNT†	14.91	69	86.36±5.74	5.11±0.32	83.22 ±17.24	7.76 ±0.87

7	Indole*	17.94	117	30.11±7.99	1.57±0.31	21.28±4.61	2.32 ±0.57
8	(*Z*)-Jasmone†	19.39	164	5.47±1.86	0.27±0.07	1.59 ±0.30	0.18 ±0.03
9	Caryophyllene*	19.93	93	14.74±4.50	0.74±0.18	2.82 ±1.22	0.21 ±0.09
10	TMTT†	21.54	69	11.09±2.89	0.68±0.18	12.13± 3.48	1.08 ±0.23
11	Unknown compound 1	21.84	149	UD #	UD	UD	UD
	Other compounds						
12	Acetonitril†	2.430	41,40	3.10±0.44	0.18± 0.02	2.19± 0.32	0.28 ±0.08
13	2-Butanone†	4.120	43	15.07±2.80	0.83± 0.10	17.01± 2.11	1.77 ±0.28
14	1-Penten-3-ol*	6.10	57	5.48±1.62	0.28± 0.06	3.02± 0.49	0.36± 0.09
15	Butanal, O-methyloxime†	6.19	41,73	0.84±0.08	0.05± 0.01	0.86± 0.17	0.09 ±0.02
16	Butanenitrile, 2 methyl†	7.10	55	2.30±0.49	0.13±0.02	2.64± 0.49	0.30 ±0.07
17	Butanenitrile, 3 methyl†	7.25	43	8.00±2.11	0.42±0.09	6.29± 1.49	0.68 ±0.14
18	Propanaloxime, 2-methyl-, syn-†	7.79	70	0.23±0.06	0.01±0.001	0.18± 0.04	0.02 ±0.01

19	Butyl acrylate†	8.47	55	1.28±0.27	0.08±0.02	1.96± 0.34	0.22 ±0.04
20	1-Octene†	8.66	43	0.43±0.04	0.03±0.001	0.49±0.10	0.07 ±0.03
21	Hexenal*	8.85	44	1.16±0.25	0.07±0.02	1.20±0.24	0.16 ±0.06
22	Butyl acetate†	9.14	43	1.10±0.32	0.06±0.02	0.64±0.14	0.07 ±0.02
23	Butyl aldoxime, 3-methyl-, syn-†	9.95	59	1.48±0.40	0.08±0.02	0.61±0.12	0.07 ±0.01
24	Butyl aldoxime, 2-methyl-, syn-†	10.00	73	0.42±0.09	0.02±0.00	0.36± 0.06	0.05 ±0.02
25	Butyl aldoxime, 2-methyl-, anti-†	10.18	59, 87	0.30±0.04	0.02±0.00	1.22± 0.71	0.23 ±0.14
26	Butyl aldoxime, 3-methyl-, anti†	10.32	59	0.34±0.08	0.02±0.00	0.24± 0.11	0.03 ±0.01
27	Amyl acetate†	11.19	43,70	1.95±0.36	0.11±0.01	1.71± 0.46	0.22 ±0.11
28	Prenyl acetate†	11.37	43, 68	0.96±0.14	0.05±0.01	0.81± 0.07	0.10 ±0.02
29	1-Octen-3-ol*	12.47	57	3.46±0.57	0.20±0.03	1.28± 0.23	0.12 ±0.01
30	3-Octanone	12.62	128	0.12±0.02	0.01±0.00	0.74± 0.27	0.10 ±0.04
31	β-Myrcene*	12.74	93	5.02±0.33	0.30±0.02	3.80± 0.53	0.37 ±0.02

32	n-Hexyl acetate*	13.04	56, 84	3.80±0.51	0.22±0.02	3.61± 0.40	0.44 ±0.09
33	(*Z*)-Linalool oxide*	14.32	59	1.37±0.26	0.08±0.02	0.90± 0.23	0.12 ±0.05
34	(*E*)-Linalool oxide*	14.57	59	2.22±0.39	0.13±0.03	0.70± 0.34	0.06 ±0.02
35	Myrtenyl acetate†	14.99	91	1.97±0.38	0.12±0.03	3.62 ±0.38	0.39 ±0.07
36	Allo-ocimene†	15.23	105, 136	1.13±0.14	0.07±0.01	0.85 ±0.17	0.08 ±0.02
37	(*Z*)-3-hexenyl isobutyrate*	15.3	67,71	2.57±0.59	0.14±0.02	1.51 ±0.24	0.20 ±0.06
38	Benzyl nitrile†	15.44	117	4.10±1.90	0.19±0.08	2.54±2.04	0.24 ±0.19
39	(*Z*)-3-hexenyl butyrate*	15.99	67	2.19±0.59	0.11±0.02	1.30 ±0.28	0.16± 0.06
40	Methyl salicylate*	16.38	120,152	1.89±1.15	0.12±0.08	1.16 ±0.41	0.10 ±0.03
41	Z-3-Hexenyl valerate*	16.70	67	2.95±0.44	0.17±0.02	0.92 ±0.24	0.08 ±0.02
42	Z-3-Hexenyl isovalerate*	16.77	67	1.72±0.21	0.10±0.01	1.11 ±0.29	0.13 ±0.04
43	Methyl anthranilate†	18.65	119	0.89±0.23	0.05±0.01	0.82 ±0.33	0.12 ±0.07
44	α-copaene†	19.22	161	1.01±0.26	0.05±0.01	0.64 ±0.14	0.08 ±0.03

45	Verbenone (C10H14O)	19.60	107, 150	1.97±0.49	0.12±0.03	1.09 ±0.23	0.12 ±0.03
46	Junipene†	19.84	94,161	1.56±0.67	0.10±0.04	3.84 ±1.30	0.51 ±0.18
47	α-Farnesene†	20.64	93	6.99±2.77	0.33±0.11	1.63 ±0.85	0.15 ±0.06
48	Isopropyl myristate†	24.25	102	0.58±0.20	0.04±0.01	0.35 ±0.09	0.04 ±0.01

***** Compounds were identified by comparison of their retention time and MS-spectra with those of authentic compounds.

† Compounds were tentatively identified by comparison of their MS-spectra with those of in the NIST02, NIST05, and Wiley libraries and in the Wageningen Mass Spectral Database of Natural Products.

‡ M/Z fragments used for quantification of volatile compounds by Thermo Xcalibur chemical station.

# UD: undetectable compound.

Table S4 Absolute and relative (%) amount of volatiles released from Lima bean plants treated with 1mM SA for 24h and then with 0.001 mM JA application for another 24h and from plants with control (tap water treatment) and 0.001mM JA applications in same time interval.

	**Chemical compound**	Retention Time	M/Z ‡ fragments	1 mM SA+Water		1mMSA+ 0.001mM JA	
				Quantities (ng / g / h)	Relative %	Quantities (ng / g / h)	Relative %
	Major compounds						
1	*Z*-3-Hexen-ol*	10.06	67	1.27±0.13	0.92±0.12	1.24±0.26	1.34±0.38
2	*Z*-3-Hexenyl acetate *	12.95	43	14.04±1.98	9.75± 1.03	10.02±1.26	10.57±1.53
3	*Z*-β-ocimene*	13.55	93	1.98±0.43	1.27±0.13	1.25±0.16	1.24±0.10
4	E-β-ocimene*	13.77	93	47.98±11.92	29.91±3.42	30.90±4.51	28.73±2.43
5	Linalool*	14.65	93	11.03±2.65	6.94±0.92	10.00±1.15	9.85±0.42
6	DMNT†	14.91	69	22.82±5.17	14.58±1.41	18.02±2.18	16.07±1.00

7	Indole*	17.94	117	4.61±1.21	2.87±0.59	4.60±0.96	4.81±1.00
8	*Z*-Jasmone†	19.39	164	UD #	UD	UD	UD
9	Caryophyllene*	19.93	93	0.55±0.04	0.41±0.06	0.68±0.14	0.70±0.12
10	TMTT†	21.54	69	4.89±0.86	3.42±0.53	3.43±0.59	3.48±0.47
11	Unknown compound 1	21.84	149	10.88±2.08	9.00±2.57	5.63±1.78	5.08±1.92
	Other compounds						
12	Acetonitril†	2.430	41,40	3.95±0.83	3.04±0.81	2.74±0.52	2.84±0.50
13	2-Butanone†	4.120	43	5.67±0.66	3.95±0.29	5.68± 0.48	5.03±0.23
14	2-Pentanone	6.22	43, 86	1.00±0.08	0.79±0.14	0.71±0.08	0.74±0.10
15	1-Octene†	8.66	43	0.37±0.04	0.29±0.06	0.40±0.05	0.41±0.05
16	Hexenal*	8.85	44	0.68±0.06	0.52±0.09	0.69±0.05	0.71±0.05
17	3-Octanone	12.62	128	0.72±0.12	0.56±0.14	0.78±0.09	0.80±0.09
18	β-Myrcene*	12.74	93	0.32±0.05	0.22±0.03	0.25±0.03	0.26±0.04

19	*E*-Linalool oxide*	14.57	59	0.49±0.04	0.36±0.04	0.57±0.07	0.58±0.06
20	Methyl salicylate*	16.38	120,152	2.96± 0.94	1.91±0.57	2.09±1.02	2.01±0.96
21	*Z*-3-Hexenyl valerate*	16.70	67	0.46±0.05	0.35±0.07	0.34±0.04	0.37±0.07

***** Compounds were identified by comparison of their retention time and MS-spectra with those of authentic compounds.

† Compounds were tentatively identified by comparison of their MS-spectra with those of in the NIST02, NIST05, and Wiley libraries and in the Wageningen Mass Spectral Database of Natural Products.

‡ M/Z fragments used for quantification of volatile compounds by Thermo Xcalibur chemical station.

\# UD: undetectable compound.

Table S5. Absolute and relative (%) amount of volatiles released from Lima bean plants treated with controls (tap water and 0.001% ethanol treatment)

	**Chemical compound**	Retention Time	M/Z ‡ fragments	Water		0.001% ethanol	
				Quantities (ng / g / h)	Relative %	Quantities (ng / g / h)	Relative %
	Major compounds						
1	*Z*-3-Hexen-ol*****	10.06	67	0.70±0.13	1.96±0.27	1.45±0.93	1.27±0.52
2	*Z*-3-Hexenyl acetate *****	12.95	43	4.65±1.03	13.05± 2.56	7.04±2.5	20.43±1.53
3	*Z*-β-ocimene*****	13.55	93	0.76±0.19	2.15±0.15	0.86±0.23	2.51±0.32
4	E-β-ocimene*****	13.77	93	3.72±0.99	10.42±1.60	3.62±1.21	10.49±2.13
5	Linalool*****	14.65	93	4.75±0.40	13.34±1.27	4.85±2.10	14.10±1.54
6	DMNT†	14.91	69	10.67±2.06	29.94±1.49	8.94±2.75	25.97±0.92
7	Indole*****	17.94	117	1.21±0.11	3.40±0.89	1.49±0.74	4.32±0.82

8	*Z*-Jasmone[†]	19.39	164	UD[#]	UD	UD	UD
9	Caryophyllene*****	19.93	93	UD[#]	UD	UD	UD
10	TMTT[†]	21.54	69	6.23±2.83	17.43±3.53	2.48±0.50	7.50±1.12
	Other compounds						
11	1-Penten-3-ol*****	6.10	57	0.72±0.14	2.02±0.52	0.44±0.10	1.27±0.18
12	Propanaloxime, 2-methyl-, syn-[†]	7.79	70	0.17±0.02	0.46±0.09	0.21± 0.10	5.03±0.23
13	Hexenal*****	8.85	44	0.67±0.04	1.89±0.11	0.83±0.08	2.41±0.71
14	3-Octanone*****	12.62	128	1.04±0.16	2.91±0.14	1.04±0.21	3.0±0.62
15	β-Myrcene*****	12.74	93	0.15±0.05	0.41±0.15	0.81±0.42	2.35±0.88
16	*Z*-3-Hexenyl valerate*****	16.70	67	0.23±0.03	0.63±0.04	0.27±0.09	0.79±0.08

***** Compounds were identified by comparison of their retention time and MS-spectra with those of authentic compounds.

[†] Compounds were tentatively identified by comparison of their MS-spectra with those of in the NIST02, NIST05, and Wiley libraries and in the Wageningen Mass Spectral Database of Natural Products.

‡ M/Z fragments used for quantification of volatile compounds by Thermo Xcalibur chemical station.

# UD: undetectable compound.
